# Petrographic and geochemical data of high alkaline basalts, Sisaket Terrain, NE Thailand

**DOI:** 10.1016/j.dib.2021.107540

**Published:** 2021-11-03

**Authors:** Vimoltip Singtuen, Sirinthorn Phajan

**Affiliations:** Department of Geotechnology, Faculty of Technology, Khon Kaen University, 123 Moo 16 Mittraphap Rd., Nai-Muang, Muang, Khon Kaen, Thailand

**Keywords:** Geochemistry, Petrology, Cenozoic basalt, Trace elements, Rare earth elements, ICP-MS

## Abstract

This data article presents mineralogical and geochemical data of high alkaline basalts in Sisaket province, the southern part of Khorat Plateau, NE Thailand. Under the polarized light microscope, the photomicrographs divided the basalts into olivine basalt and alkaline basalt with four textures: aphanitic, porphyritic, vesicular, and diabase. These basaltic rocks comprise olivine microphenocrysts associated with labradorite-anorthite (An_66-94_), clinopyroxene, opaque minerals groundmass. In addition, nepheline is only found in alkaline basalt as groundmass. Major oxides (Na_2_O+K_2_O and SiO_2_) suggest that Sisaket basalts are basalt, basanite, trachy basalt, and basaltic trachy-andesite. High ratio Nb/Y and low Zr/Ti classify these basalts as alkaline basalt and basanite.

## Specifications Table

**Table:** 

Subject	Earth and Planetary Sciences
Specific subject area	Geochemistry and Petrology
Type of data	table, image, graph
How data were acquired	ZEN core Imaging Software, linking ZEISS imaging and microscope solutions for petrographic study (supplementary file). A furnace (at 1000 °C) for loss on ignition (LOI) analysis. Phillip-MagixPro PW 2400 Wavelength Dispersive X-Ray Fluorescence spectrometer measure major oxides. Inductively coupled plasma Mass Spectrometer (ICP-MS) and inductively coupled plasma Atomic Emission Spectrometer (ICP-AES) analyze trace elements and rare earth elements.
Data format	‘raw’ and ‘analyzed’
Parameters for data collection	rock samples were least-altered representativeness and no secondary mineral replacement.
Description of data collection	Basalt samples were collected from the outcrop by different features, labelled their location on the map, and transferred to Khon Kaen University. Representatives were cut and made as thin sections for studying petrography and microphotograph analysis. The least-alters were posted to Chiang Mai University to grind them as 200 mesh and analyze major oxides, including SiO_2_, TiO_2_, Al_2_O_3_, Fe_2_O_3_t, MgO, CaO, Na_2_O, and K_2_O. Loss on ignition (LOI) was analyzed in a furnace (1000 °C for 12 h) at the Department of Architecture, Khon Kaen University. In addition, 500 grams of each sample were posted to China for measuring trace elements and rare earth elements concentrations present in ppm.
Data source location	latitudes 14^o^15’N to 15^o^N and longitudes 104^o^22’E to 104^o^45’E Khun Han and Kantharalak Districts, Sisaket Province, Thailand Primary data sources of geologic maps: www.dmr.go.th/dmr_data/downloadmap/geologicmap/ND48-6.zip[8]www.dmr.go.th/download/pdf/NorthEast/srisaket.pdf[9]
Data accessibility	Raw data are provided with this article as supplementary materials.

## Value of the Data

• These data could be advantages for the department of mineral resources, department of primary industries and Mines, and other universities or academic centres related to geological sciences, which need these to study petrochemistry and tectonics evolution.
• The database can be linked with other applied sciences for increasing research values such as remote sensing, geophysics, and engineering geology.
• The geochemical data of these rocks could help the department of agriculture develop their (parent rock/soil) research and agricultural area-based planning.
• The database could be compared with the data from other experiments for further insights and reducing analysis costs. It could be the chemical data standard of rock for other Thai institutions.
• In humid climates or tropical zone such as Thailand, *in situ* weathering of high alkaline basalt is almost the main source of potential soil, therefore, the continuous monitoring of the quality of these valuable resources is very necessary for agricultural industries.

## 1 Data Description

Thailand, mainland south-eastern Asia, presents complex geological setting by the collision of the Indochina and SIBUMASU Terrain. This region is composed of many rocks, including metamorphic, sedimentary, plutonic, and volcanic rocks. According to various volcanic rocks, Thai's tectonic evolutions are interpreted during Silurian to Quaternary, especially the youngest basalts. Cenozoic basalts of Thailand erupted into small bulbs [1] scattered in Chiang Rai, Lampang, Phrae, Lopburi [2,3], Phetchabun [4,5], Kanchanaburi, Surin, Ubon, Ratchathani, Chanthaburi, Prachinburi, Trat Provinces [6] and the southern edge of the Korat Plateau, including Nakhon Ratchasima, Buriram, and Sisaket [1,6].

**Fig. 1:**
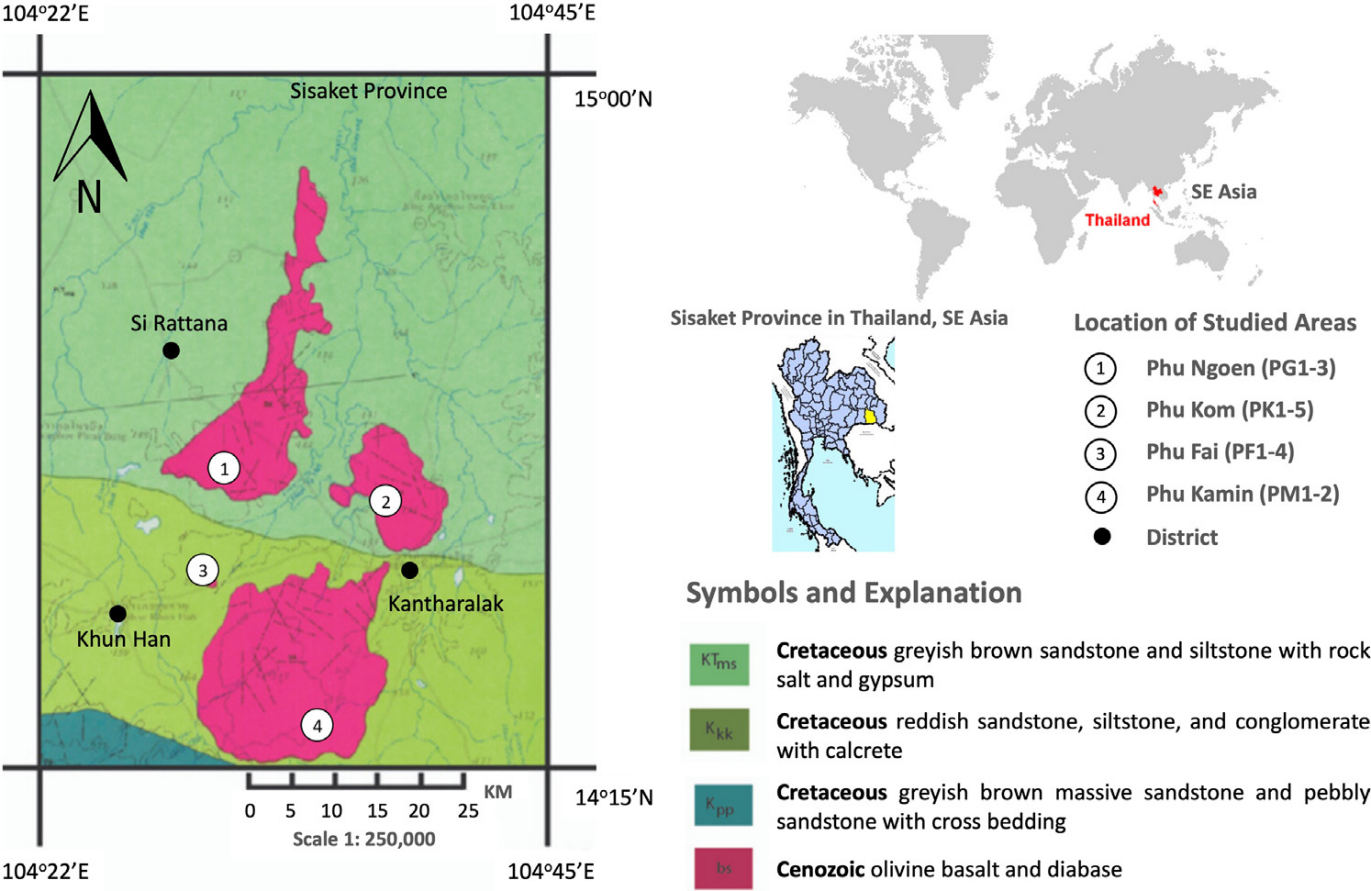
Geologic map of Sisaket Province, NE Thailand [8,9] and location of the study areas, which consist of Phu Ngoen (PG1-3), Phu Kom (PK1-5), Phu Fai (PF1-4), and Phu Kamin (PM1-2).

Sisaket province is located in the southern part of the Khorat Plateau, part of Indochina Terrain. This city demonstrates many geologic resources related to Cenozoic basalt, i.e., gemstone (sapphire), agricultural areas planted volcano Durian. Sisaket Cenozoic basalts (bs) erupted and flowed on the Cretaceous to Tertiary sedimentary rocks of Khorat Group: Khok Kruat (K_kk_) and Mahasarakham (KT_ms_) Formations [7], [8], [9] as shown in Fig. 1.

The geologic map of Choam Khsan (1: 250,000) and Sisaket Province (1: 1,000,000) are provided as primary data sources [8,9]. There are three main volcanic areas distributed in two districts (Khun Han and Kantharalak) of Sisaket province includes (1) Phu Ngoen, (2) Phu Kom, (3) Phu Fai, and (4) Phu Kamin. The basalts in Phu Ngoen and Phu Fai were classified as alkaline basalts with high Na_2_O [1]. On the other hand, the Phu Fai diabase was classified as mugearite and Phonotephrite that intruded during 3.28 ± 0.28 ma [10,11].

**Fig. 2:**
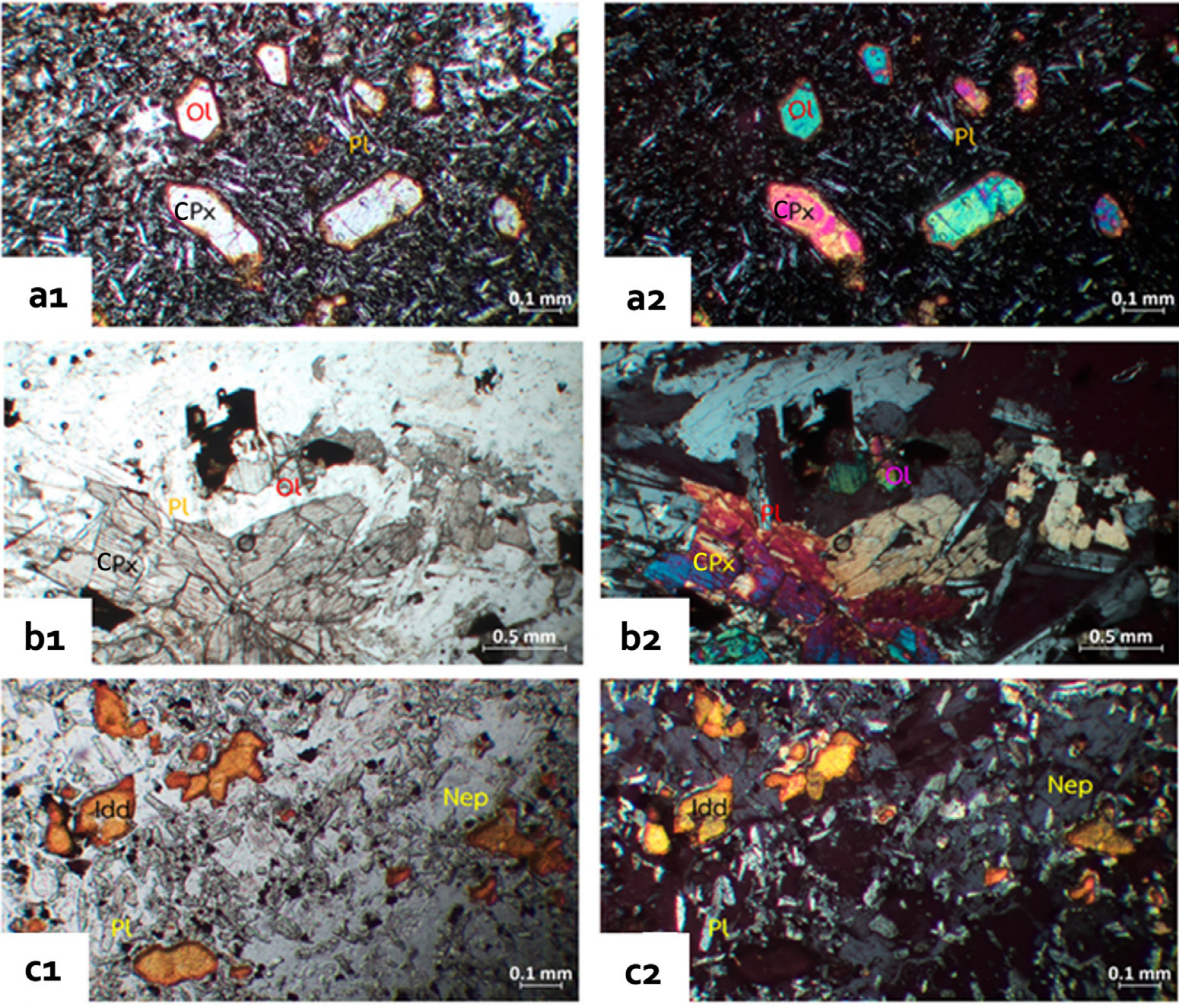
Photomicrographs by polarizing microscope (a) Phu Kom olivine basalt with porphyritic texture, (b) Phu Fai diabase with ophitic/ subophitic texture, and (c) Phu Ngoen alkaline basalt with a vesicular texture and microphenocrysts, presenting in plane-polarized light (1) and cross-polarized light (2); Ol: olivine, Pl: plagioclase, CPx: clinopyroxene, Nep: nepheline, Idd: iddingsite.

Based on their textures under the polarized light microscope, the basalts can be divided into four groups: aphanitic, porphyritic, vesicular, and diabase texture. There are two series of basaltic rocks comprising olivine basalt and alkali basalt. Petrographic features of the studied basalts are illustrated in Fig. 2. Photomicrographs of studied basalts under the polarized light microscope are provided as a supplementary file.

Table 1 presents the mineral composition of studied basalts by petrographic analysis. Olivine basalts present aphanitic, porphyritic, and diabase textures, comprising labradorite-bytownite (An_66-85_), olivine, clinopyroxene, and ilmenite. Microphenocrysts (9.02–21.19 %) consist of olivine exhibited 0.09–0.45 mm euhedral-subhedral crystals and highly altered to iddingsite. These olivine basalts are distributed in four areas: Phu Fai (PF), Phu Ngoen (PG), Phu Kom (PK), and Phu Fai (PF). Olivine basalt also shows diabase texture with diameters of 0.1–0.475 mm or, more specifically, subophitic/ophitic texture found only in samples from the Phu Fai shallow intrusion.

**Table 1:** Petrographic analysis for mineral composition.

Min	PF1	PF2	PF3	PF4	PG1	PG2	PG3	PK1	PK2	PK3	PK4	PK5	PM1	PM2
**groundmass (%)**														

Pl	81.42	65.99	70.92	65.28	57.48	-	55.46	47.81	66.85	59.70	62.01	47.74	24.80	47.64
Ol	3.98	15.62	3.80	14.81	11.32	-	0.27	7.93	-	0.75	0.46	0.41	0.82	1.99
CPx	9.29	13.85	18.34	13.89	12.39	-	0.27	6.68	0.27	9.70	-	-	10.08	0.74
Opa	5.31	4.53	6.94	6.02	11.97	-	22.95	10.23	13.59	12.44	17.39	22.22	17.44	8.68
Idd	-	-	-	-	6.84	0.65	12.02	9.39	0.82	2.74	2.75	8.44	-	0.74
Nep	-	-	-	-	-	42.21	-	-	-	-	-	-	3.81	29.28
total	100	100	100	100	100	42.86	90.98	82.05	81.52	85.32	82.61	78.81	79.84	89.08

**phenocryst (modal%)**														

Ol						10.17	-	9.39	10.60	9.45	6.41	13.79	6.81	-
CPx						4.76	8.74	0.84	6.52	4.98	3.89	4.73	11.99	-
Pl						29.65	-	-	-	-	-	2.67	-	-
Idd						-	-	7.10	-	-	6.64	-	-	9.93
Opa						12.55	0.27	0.63	1.36	0.25	0.46	-	1.36	0.99
total						57.14	9.02	17.95	18.48	14.68	17.39	21.19	20.16	10.92

Min: mineral composition.

Pl: plagioclase, Ol: olivine, CPx: clinopyroxene, Opa: opaque mineral, Idd: iddingsite, Nep: nepheline.

Alkaline basalts with porphyritic and vesicular texture are distributed in the Phu Ngoen area. Microphenocrysts (10.92–57.14%) are made up mainly of olivine (occurred as 0.02–0.2 mm across), which presents euhedral-subhedral crystals. These olivines highly altered to iddingsite and other phyllosilicates (i.e., serpentine/chlorite). The groundmass comprises bytownite-anorthite (An_81-94_), nepheline, olivine, clinopyroxene, ilmenite, and iddingsite.

**Fig. 3:**
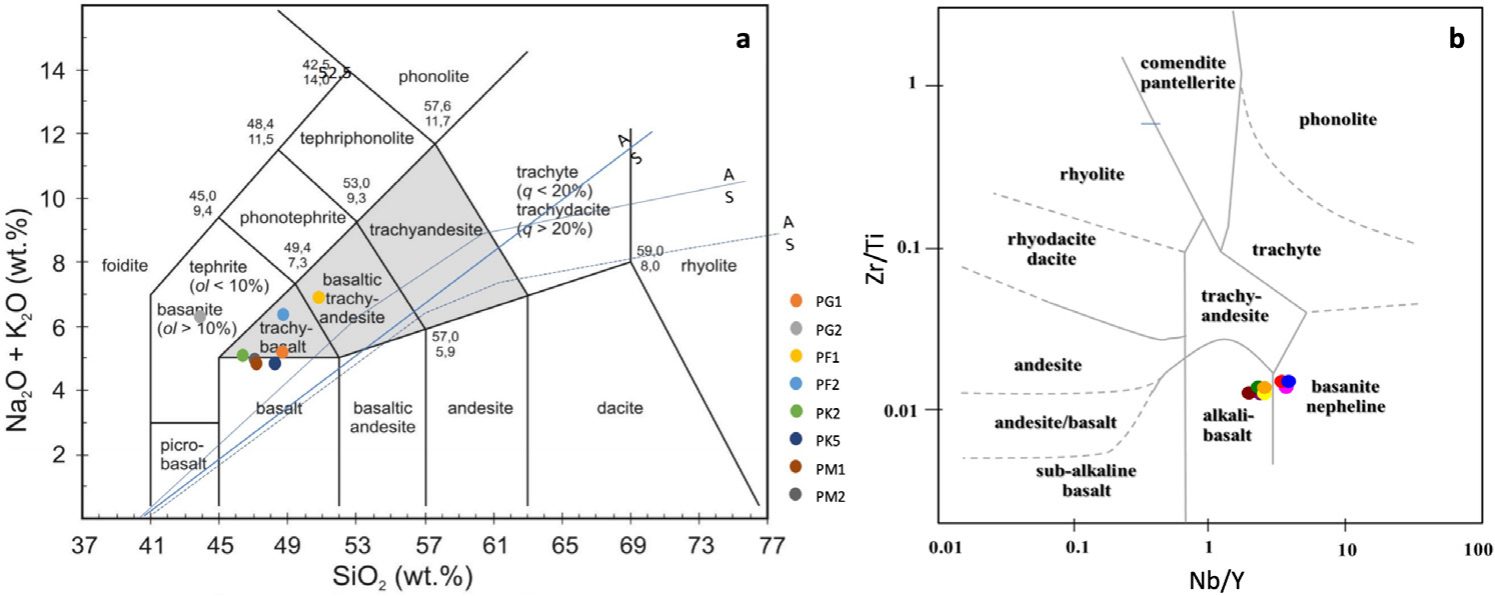
Geochemical analysis of Sisaket Basalt (a) TAS diagram, nomenclature by Na_2_O+K_2_O relates to SiO_2_ (diagram [12,13]), (b) Trace diagram plotted Zr/Ti versus Nb/Y (diagram [14]).

Based on petrographic data, eight least-altered basalts were selected for geochemical analysis, including major oxides (Table 2) and trace elements as well as rare earth elements (REE), showing in Table 3. The other major oxides of high alkaline basalt in the Sisaket area include 46.54–50.87 wt.% SiO_2_, 1.29–1.71 wt.% TiO_2_, 16.06–19.88 wt.% Al_2_O_3_, 6.33–11.25 wt.% Fe_2_O_3_t, 5.62–11.07 wt.% MgO, 7.63–10.17 wt.% CaO, 2.31–4.01 wt.% Na_2_O, and 1.08–3.20 wt.% K_2_O. Major oxides plotted in the TAS diagram suggest that Sisaket basalts rank in basalt, basanite, trachy basalt, and basaltic trachy-andesite, as present in Fig. 3a. Moreover, these basalts present high LREEs: 28.2–85.9 ppm. La, 51.10–147.00 ppm. Ce, 6.42–10.80 ppm. Pr, and 24.60–64.20 ppm. Nd, with a very high content of Zr (168–239 ppm.). According to high ratio Nb/Y (2.183–3.645) and low Zr/Ti (0.013–0.015), these basalts were classified as alkaline to high alkaline basalts or basanite (Fig. 3b).

**Table 2:** Whole-rocks analysis for major oxides (by XRF) and LOIs.

	Rock Samples							
Major Oxides (wt%)	PF1	PF2	PG1	PG2	PK2	PK5	PM1	PM2
SiO_2_	50.87	48.52	48.75	43.81	46.54	48.25	47.26	47.24
TiO_2_	1.65	1.47	1.29	1.71	1.40	1.30	1.45	1.44
Al_2_O_3_	19.88	17.98	17.87	16.06	17.62	17.74	17.62	17.60
Fe_2_O_3_	6.33	6.98	7.32	9.98	11.25	8.32	9.02	9.02
MgO	5.62	9.76	11.07	10.32	9.81	10.82	9.91	9.90
CaO	7.19	8.07	7.94	10.17	7.63	8.16	8.93	8.93
Na_2_O	3.63	3.46	2.56	4.01	2.31	3.35	3.78	3.84
K_2_O	3.20	2.94	2.64	2.25	2.79	1.47	1.08	1.09
MnO	0.04	0.12	0.07	0.04	0.08	0.10	0.06	0.07
P_2_O_5_	0.83	0.69	0.48	1.66	0.57	0.49	0.88	0.89
LOI	1.78	1.51	1.62	2.35	1.22	1.77	2.67	2.63

Major Elements (wt%)								

Ti	1.68	1.43	1.35	1.58	1.39	1.35	1.36	1.3
Mg	2.19	3.75	4.51	3.9	3.92	4.07	4.03	4.21
K	2.3	2.3	2.4	1.8	2.4	1.0	1.0	1.3
Fe	7.03	7.08	8.23	9.92	8.86	9.47	9.03	8.99
Ca	5.4	5.2	6.1	6.9	5.5	6.2	6.0	6.1
Al	9.83	9.2	8.83	7.26	8.68	8.97	8.18	7.88

**Table 3:** Whole-rocks analysis for trace elements and rare earth elements (ppm) by ICP-MS and ICP-OES.

	Rock Samples							
Trace Elements and REEs (ppm)	PF1	PF2	PG1	PG2	PK2	PK5	PM1	PM2
La	34.2	27.9	28.2	85.9	31	30.1	52.4	50.3
Ce	68.30	54.70	51.10	147.00	54.60	51.50	90.20	82.80
Pr	9.11	7.31	6.42	17.50	6.76	7.02	10.80	10.10
Nd	36.60	29.10	24.60	64.20	26.3	27.30	40.40	36.60
Sm	8.60	7.80	6.70	13.30	5.50	7.80	9.20	8.30
Eu	2.75	2.27	2.1	4.1	2.05	2.17	2.9	2.64
Gd	6.42	5.39	4.89	10.1	4.9	4.8	6.65	6.36
Tb	0.90	0.80	0.74	1.33	0.77	0.84	1.00	0.99
Dy	4.55	3.77	3.94	6.06	4.03	4.29	4.98	4.63
Ho	0.73	0.67	0.71	0.95	0.71	0.68	0.81	0.8
Er	1.84	1.62	1.79	2.24	1.76	1.92	2.02	1.86
Tm	34.2	27.9	28.2	85.9	31	30.1	52.4	50.3
Yb	1.20	1.00	1.30	1.30	1.20	1.20	1.40	1.30
Th	3.60	3.10	4.20	8.20	4.00	3.80	6.20	6.00
Ta	3.60	3.70	4.50	7.40	4.40	3.80	5.70	6.00
Nb	50.0	44.0	52.0	109.0	57.0	50.0	77.0	72.0
P	3900	3500	2900	7800	2900	2500	4200	5000
Zr	212.0	177.0	168.0	239.0	176.0	174.0	191.0	190.0
Hf	5.00	5.00	4.00	6.00	4.00	4.00	4.00	4.00
Y	22.90	18.50	21.40	29.90	21.90	19.50	22.50	21.70
Ba	485	397	400	576	575	347	445	422
Ce	68.3	54.7	51.1	147	54.6	51.5	90.2	82.8
Co	29.6	36.9	41.8	38.3	47.8	43.8	41.3	46.2
Cr	70	172	183	131	224	266	293	309
Cs	0.7	0.4	0.5	0.8	0.7	0.6	0.8	0.8
Cu	55	55	70	71	70	51	68	70
Ga	30	29	27	29	27	26	28	29
Lu	0.18	0.17	0.21	0.2	0.2	0.21	0.2	0.21
Mn	841	910	1252	1422	1100	980	1230	1394
Mo	3	2	3	7	4	4	4	4
Ni	24	47	104	94	133	149	158	174
Rb	36.8	32.0	33.3	68.3	32	45	50.4	48
Sc	14	18	22	13	19	21	19	18
Sr	1030	1006	733	1435	911	756	981	950
Tb	0.9	0.8	0.74	1.33	0.77	0.84	1.00	0.99
U	1.13	1.0	0.86	2.13	1.32	1.19	1.78	1.78
V	149	136	165	161	163	160	163	189
W	87	111	58	32	107	68	47	42
Zn	94	92	96	149	96	92	110	125

## 2 Experimental Design, Materials and Methods

The samples were collected from the basalt outcrops in latitudes 14^o^15’N to 15^o^N and longitudes 104^o^22’E to 104^o^45’E of Sisaket province: (1) Khun Han District consists of Wat Pa Si Somboon Phudin (Tha), Phu Ngoen Stone Mill, Phu Fai, and Sila Phu Fai Limited Partnership, (2) Kantharalak District comprise Phu Fai, Wat Phu Din Daeng, and Phu Din Daeng Rock Pit (Table 4). Sample PG1-3 were collected from Phu Ngoen, while PF1-4 were representative samples of Phu Fai. In addition, sample PK1-5 were taken from Phu Kom, while PM1-2 were from Phu Kamin. Outcrops always exhibit as road-cut (PF2-4 and PG1) and open-pit mining (PG2-3, PK5, and PM1-2) with sandstone xenoliths, excluding PF1, PG1, PK1-4 that were presented from *in situ* rocks at small hills.

**Table 4:** Location, sample number, character of outcrops and rock samples.

Location	Sample	Place	Outcrop	Rock Texture
14^o^38’26.51” N 104^o^29’34.55” E	PF1-PF4	Wat Prasat Phu Fai Temple, Tambon Phu Fai, Khun Han District	Road-cut outcrop and *in situ* rock at small hill	Diabase
14^o^42’35.40” N 104^o^30’30.31” E	PG1	Wat Si Sombun Phu Din Temple, Tambon Phu Fai, Khun Han District	*in situ* rock at small hill	Aphanitic
14^o^42’51.46” N 104^o^30’32.94” E	PG2-PG3	Phu Ngoen Post-Mining Area, Tambon Phu Fai, Khun Han District	Open-pit mining + sandstone xenolith	Vesicular
14^o^41’02.67” N 104^o^38’26.27” E	PK1-PK4	Wat Phu Din Daeng Temple, Tambon Nam Om, Kantharalak District	*in situ* rock at small hill	Aphanitic
14^o^40’43.21” N 104^o^38’31.64” E	PK5	Phu Ngoen Post-Mining Area, Tambon Nam Om, Kantharalak District	Open-pit mining	Vesicular
14^o^34’31.53” N 104^o^31’58.74” E	PM1-PM2	Sirisin-Phu Kamin Mining, Tambon Phran, Khun Han District	Open-pit mining + sandstone xenolith	Aphanitic

Fourteen samples were collected from the study area and made thin sections for petrographic studying with the polarized light microscope. Photomicrograph analysis was done by ZEN core Imaging Software, linking ZEISS imaging and microscope solutions at the Department of Geotechnology, Khon Kaen University.

Loss on ignition (LOI) was analyzed by heating a platinum crucible containing a 1.0 g sample (measure three-time for each sample) in a furnace at 1000 °C for 12 h at the Department of Architecture Khon Kaen University.

For loss on ignition (LOI) content, the first step of the parameter is a crucible and rock powder weight before putting them to the furnace. Calculate loss on ignition by weight of rock powder before and after flaming as follow:

$$\text{LOI}\;\left(g\right)=W_{\text{sumbef}}-W_{\text{sumaft}}=W_{\text{rockbef}}-W_{\text{rockaft}}$$

$$\text{LOI}\;\left(\%\right)=\left(\text{LOI}\;\left(g\right)/W_{\text{rockbef}}\right)\;\times \;100\%$$

Where

LOI = loss on ignition
W_sumbef_ = weight of crucible with rock sample before heating
W_sumaft_ = weight of crucible with rock sample after heating
W_rockbef_ = weight of rock sample before heating
W_rockaft_ = weight of rock sample after heating

The geochemistry was studied using a Phillip-MagixPro PW 2400 Wavelength Dispersive X-Ray Fluorescence spectrometer at the Department of Geological Sciences, Chiang Mai University, for analyzing major elements (SiO_2_, TiO_2_, Al_2_O_3_, Fe total as Fe_2_O_3_, MnO, MgO, CaO, Na_2_O, K_2_O, and P_2_O_5_). The eight least-alters were selected for their trace elements (Rb, Sr, Zr, Y, Nb, Ni, Cr, V, Sc, Hf, Th, and Ta) and rare earth elements (La, Ce, Pr, Nd, Sm, Eu, Gd, Tb, Dy, Ho, Er, Tm, and Yb). These samples (less than 200 mesh/75 microns) were analyzed using a Sodium Peroxide Fusion combined with inductively coupled plasma Mass Spectrometer (ICP-MS) and inductively coupled plasma Atomic Emission Spectrometer (ICP-AES) at the SGS-CSTC Standards Technical Services Co., Ltd., China. Three fluxes, sodium carbonate, sodium peroxide, and sodium hydroxide, are potent combinations. Low sample/flux ratios combined with a proper dilution yield a solution with acceptable total dissolved solids levels for analysis. The fusion of this sample preparation procedure takes place at low temperatures (about 500 °C), which prevents the loss of volatile components.

## Ethics Statement

This data article is the authors’ original work, which has not been previously published elsewhere. In addition, the data reflect the authors’ own research and analysis truthfully and completely. On the other hand, all sources used are correctly disclosed (correct citation).

## CRediT Author Statement

**Vimoltip Singtuen:** Conceptualization, Methodology, Data Analysis, Writing – Original draft preparation, Visualization, Investigation, Validation, Writing – Review & editing; **Sirinthorn Phajan:** Formal analysis, Petrography, and Loss on Ignition.

## Declaration of Competing Interest

The authors declare to have no known competing financial interests or personal relationships which have or could be perceived to have influenced the work reported in this article.

## Appendix Supplementary materials

Image, application 1

Image, application 2
