# Supplementary figures and images for: Petrographic and geochemical data of high alkaline basalts, Sisaket Terrain, NE Thailand
Source: Data Brief. 2021 Nov 3;39:107540. doi: 10.1016/j.dib.2021.107540 (PMC8602002; doi:10.1016/j.dib.2021.107540)

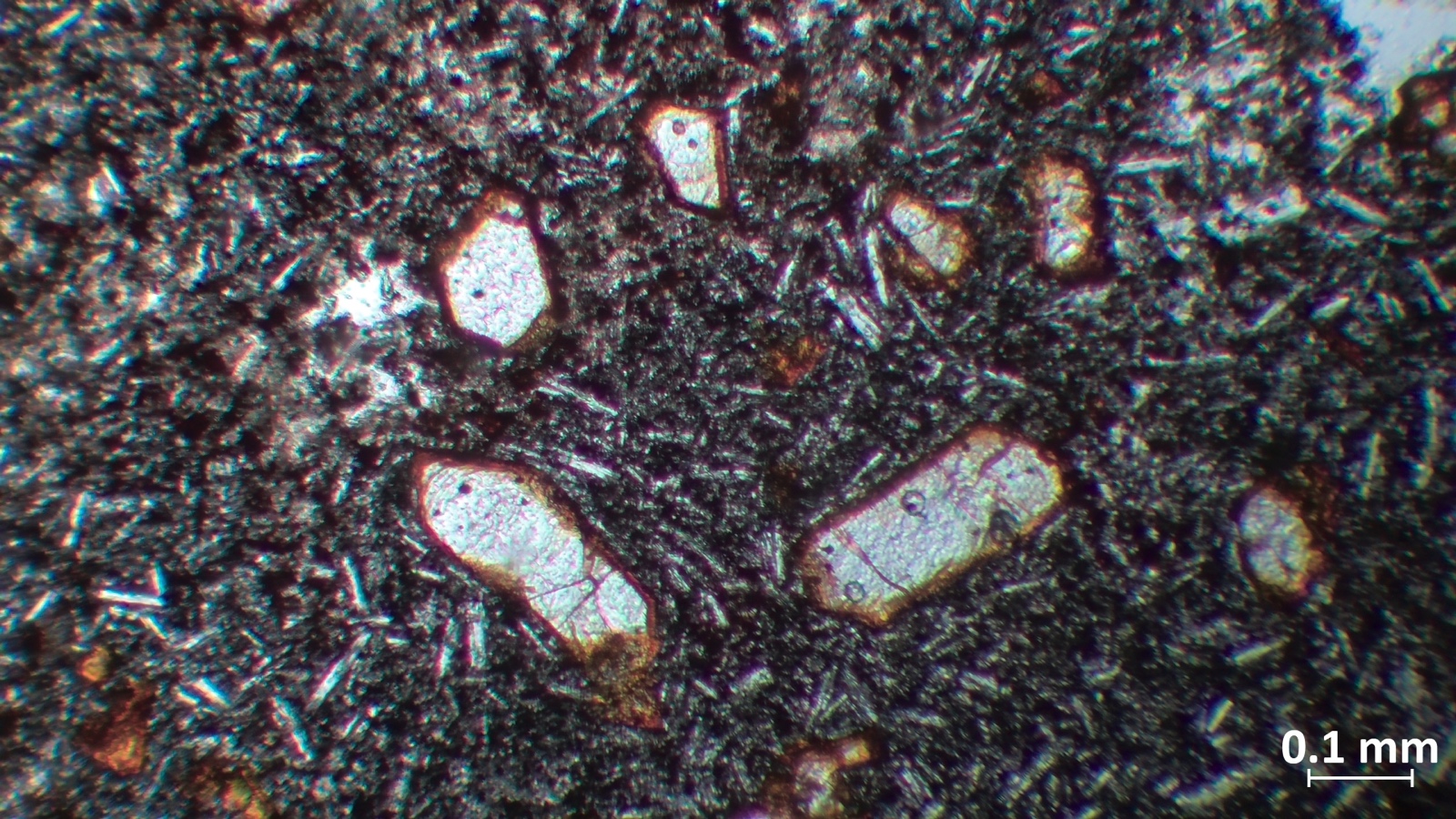


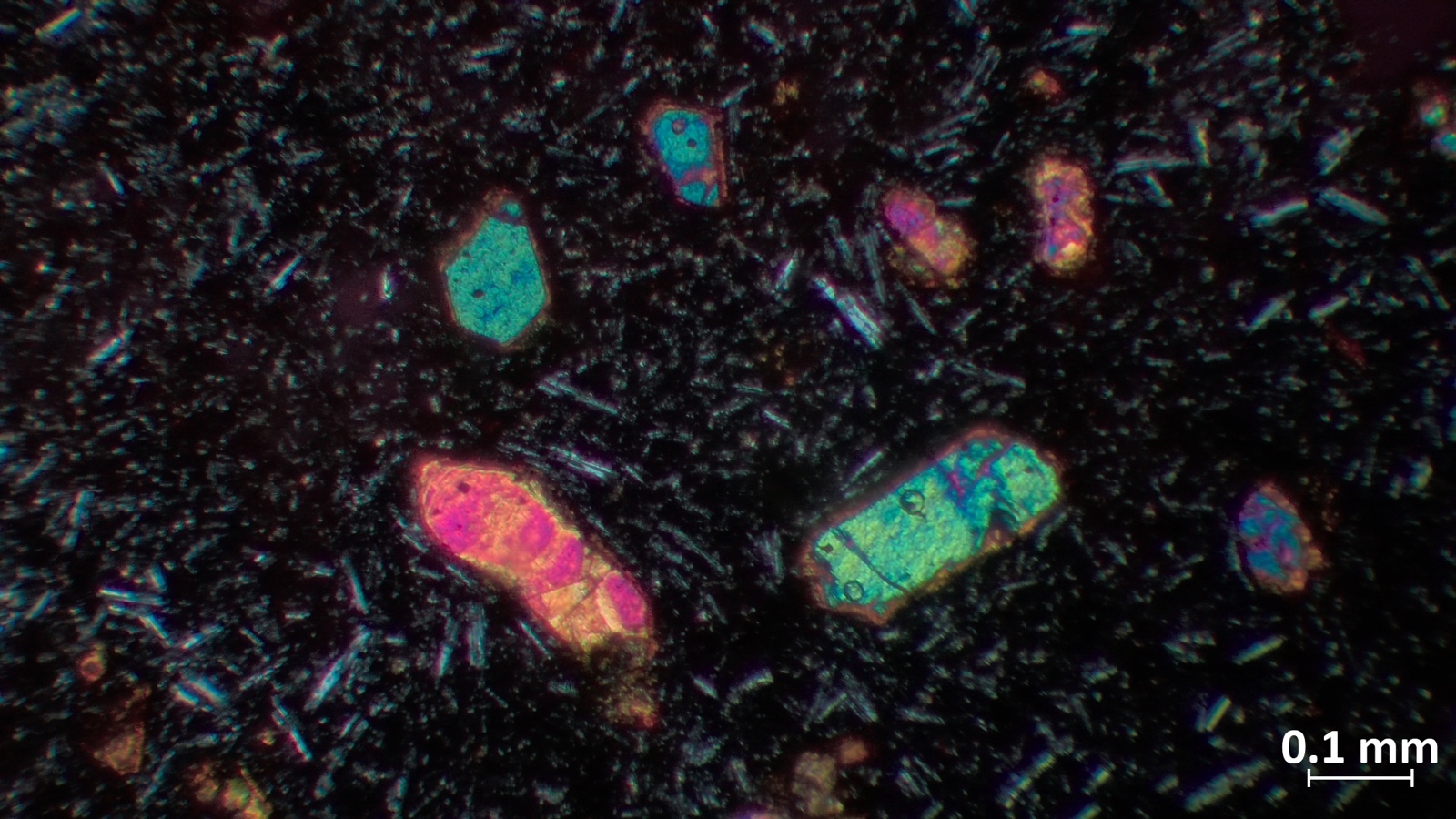


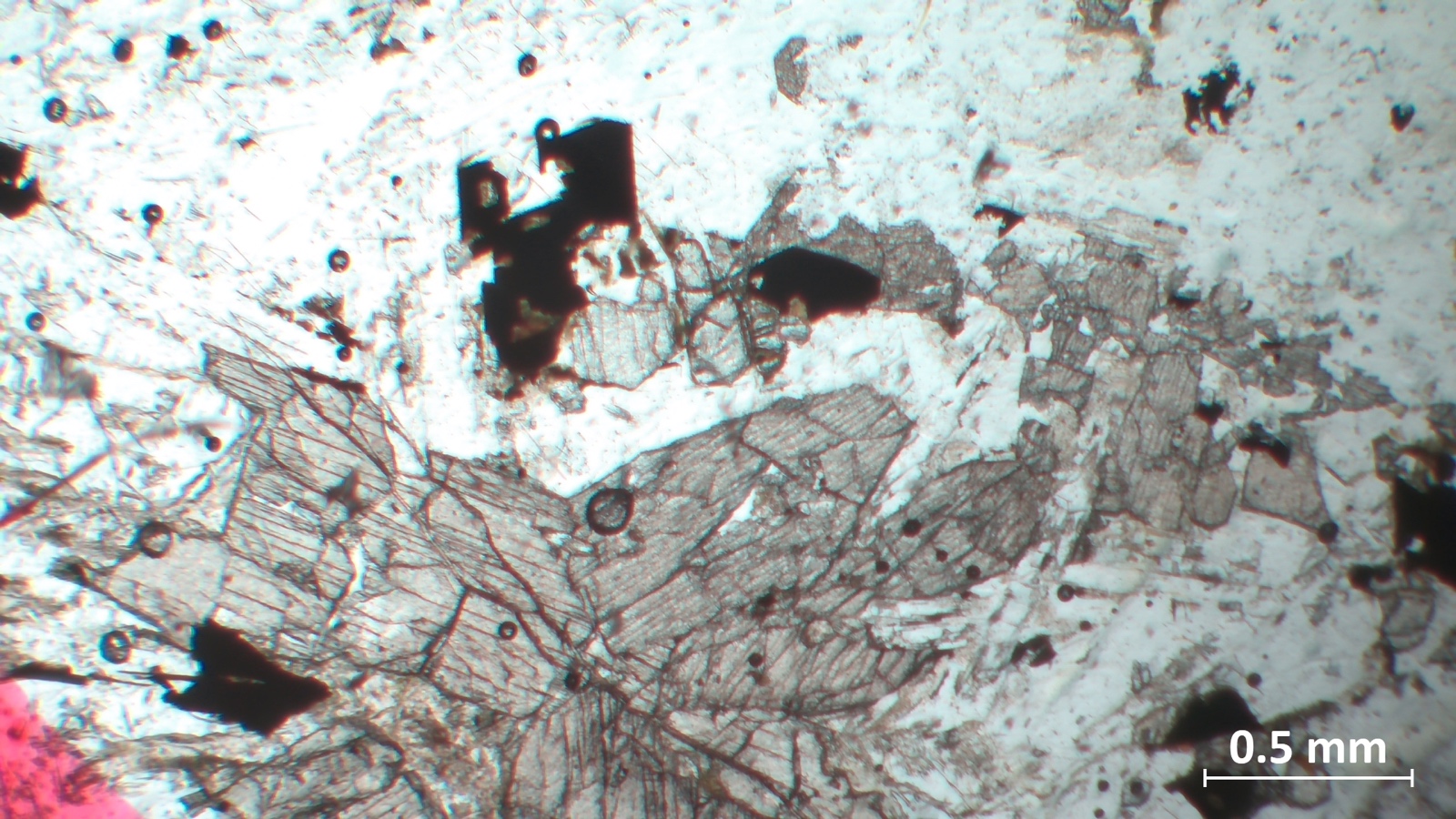


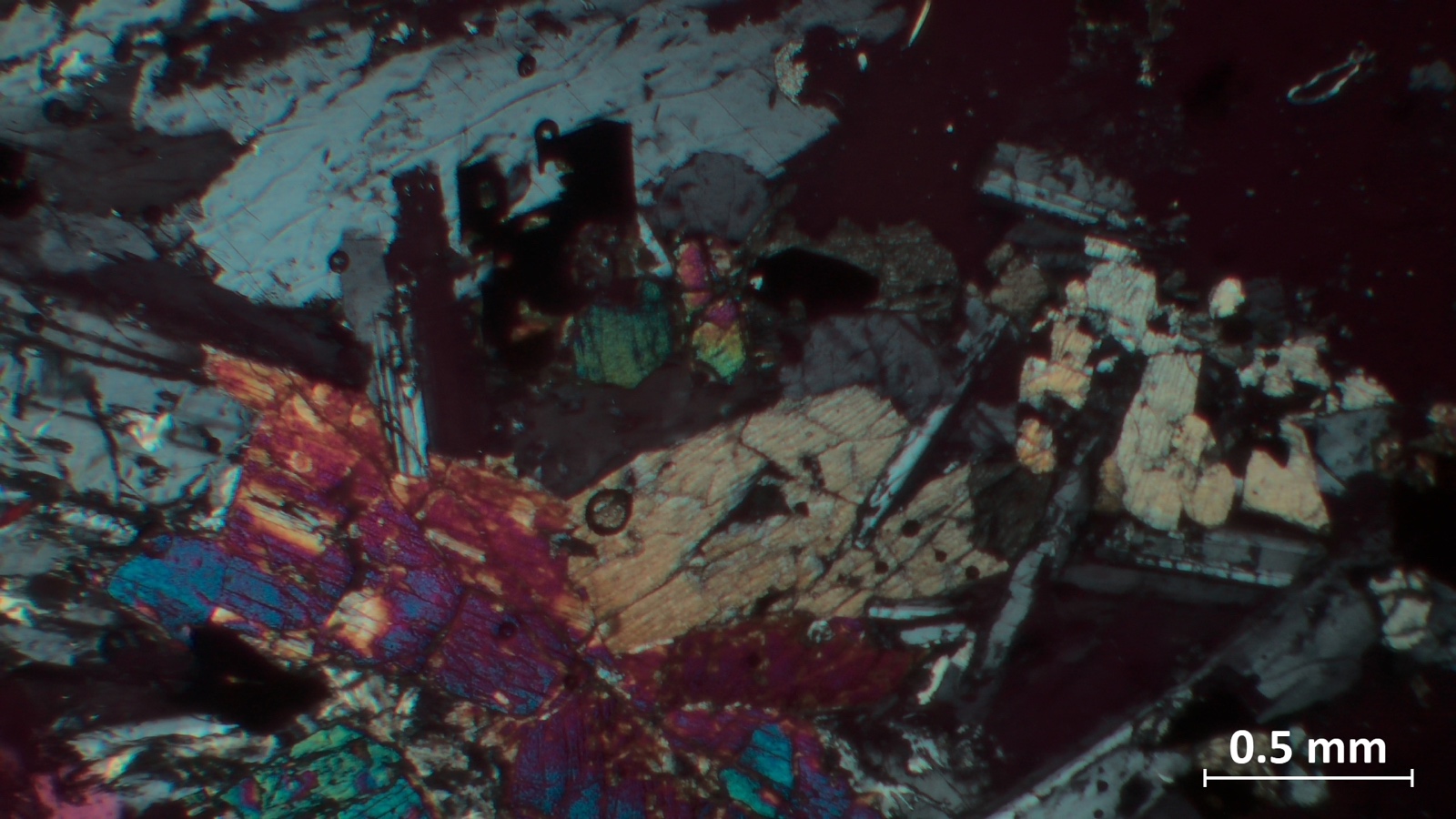


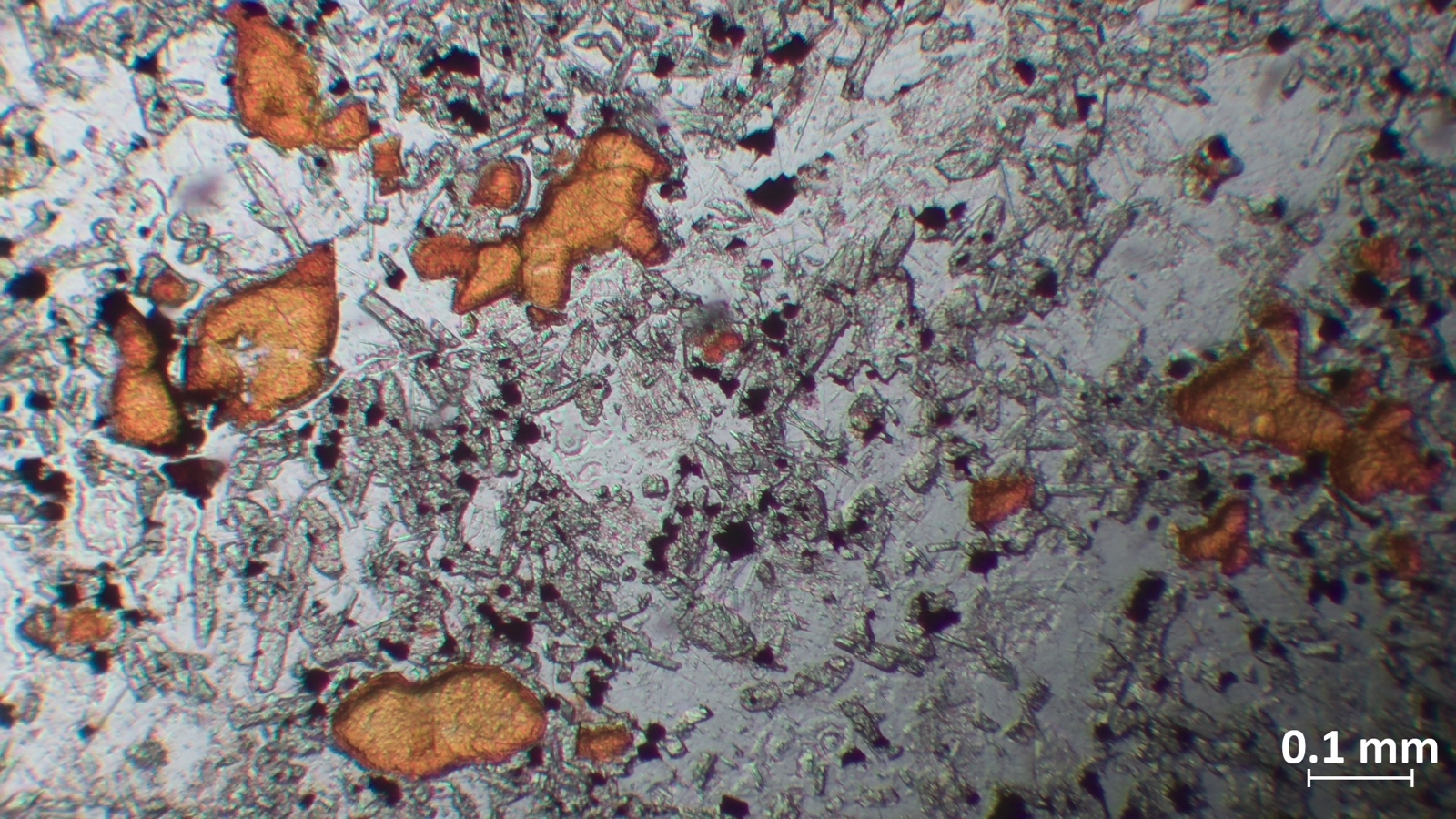


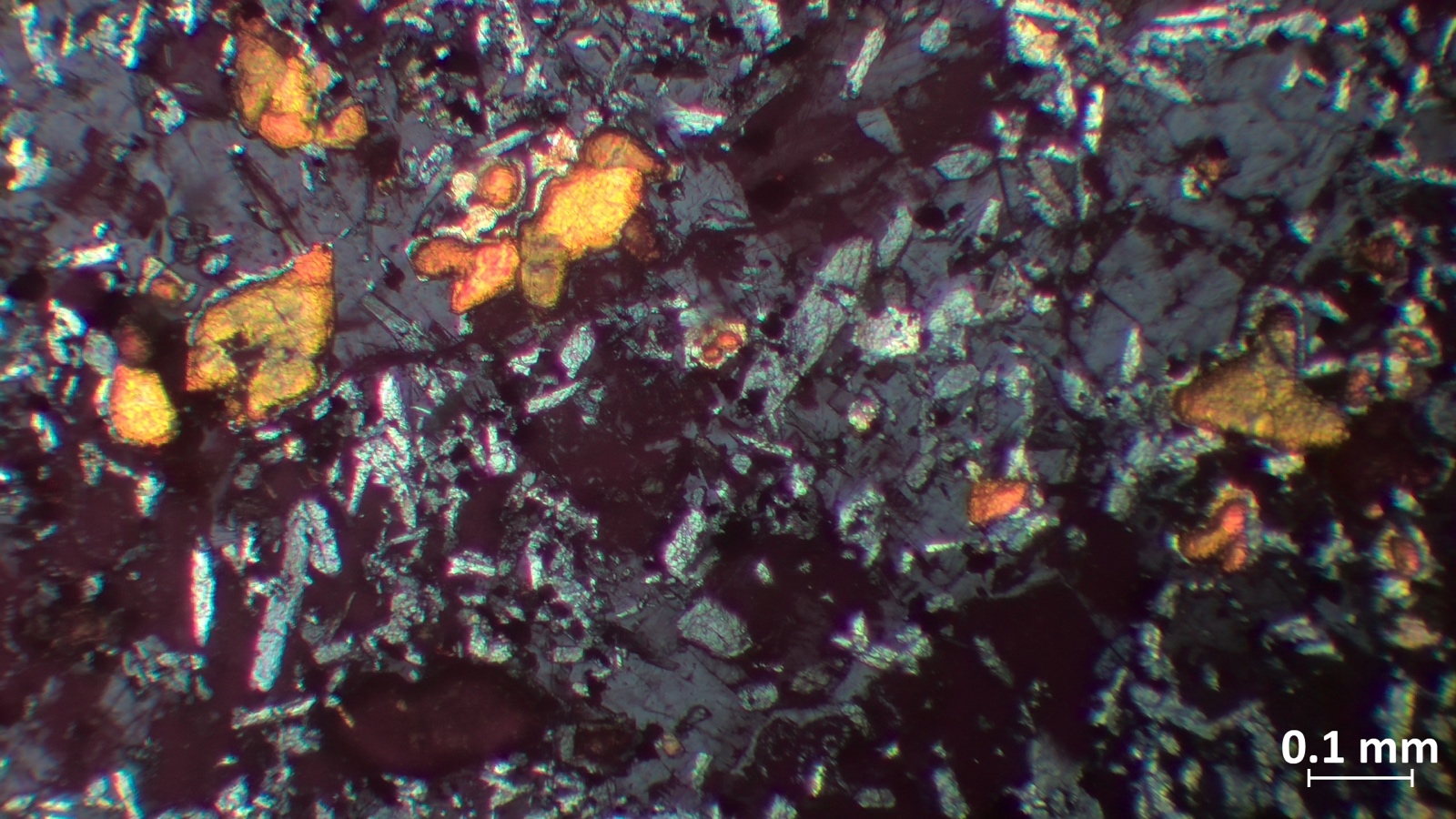


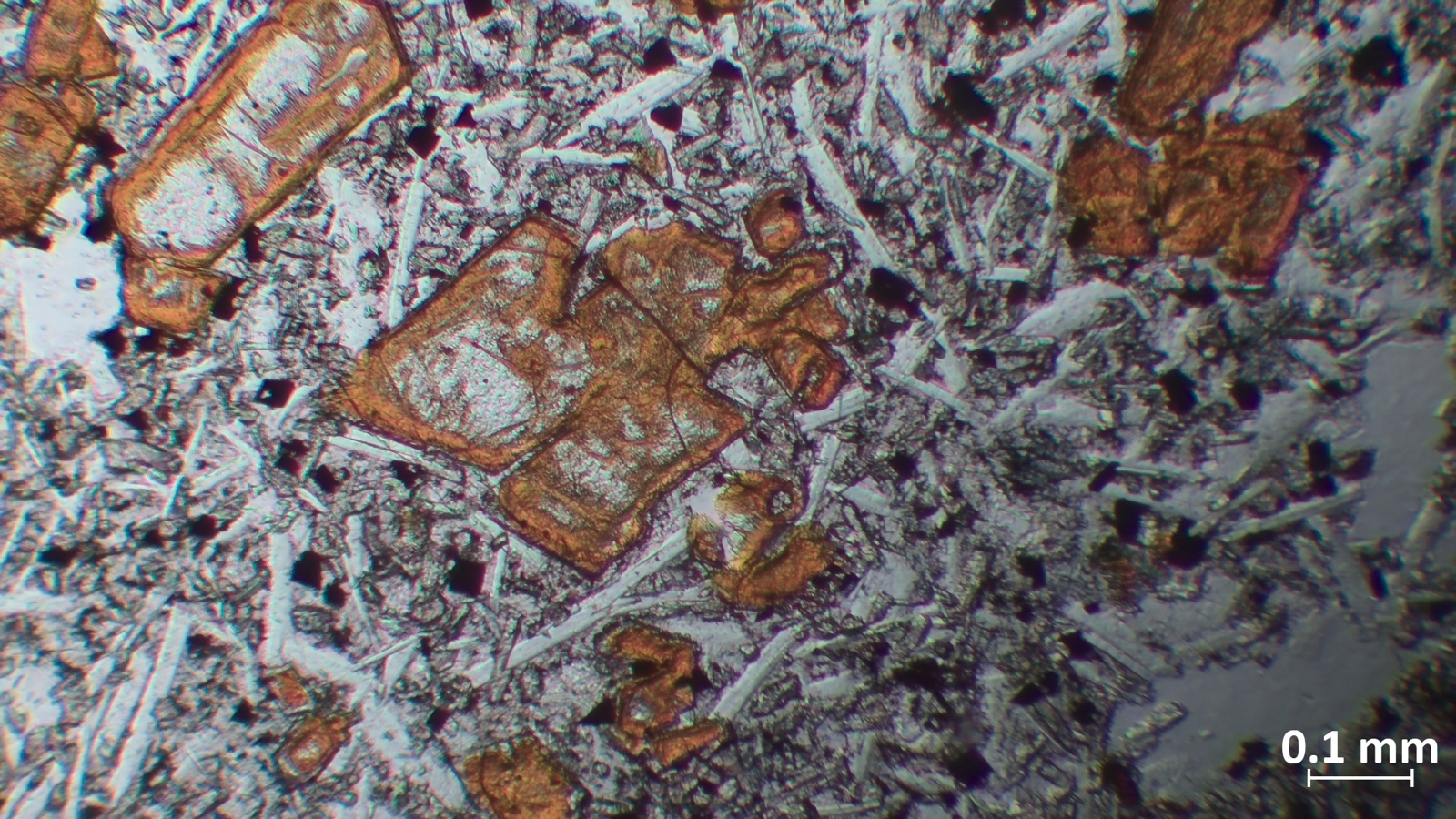


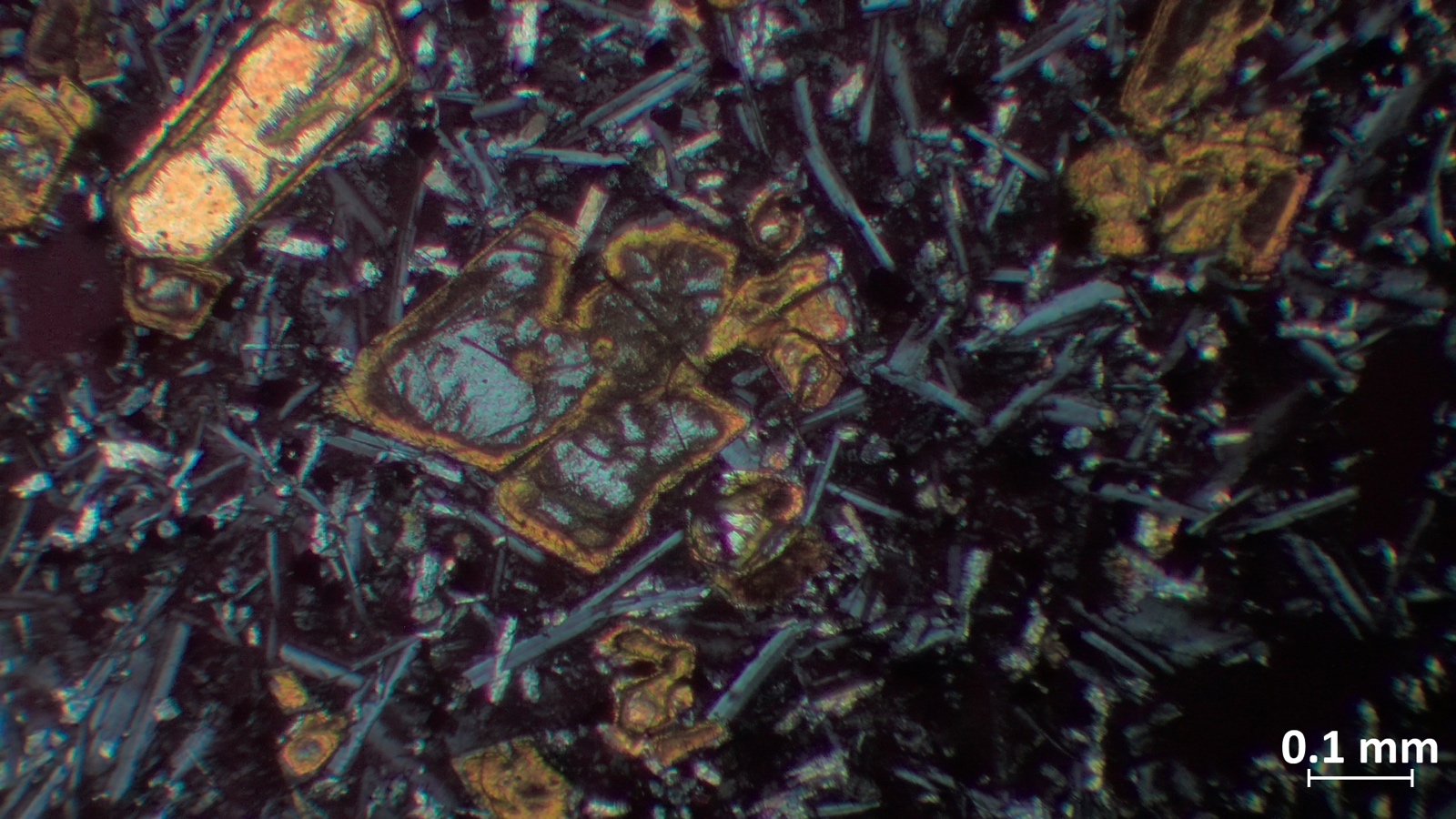


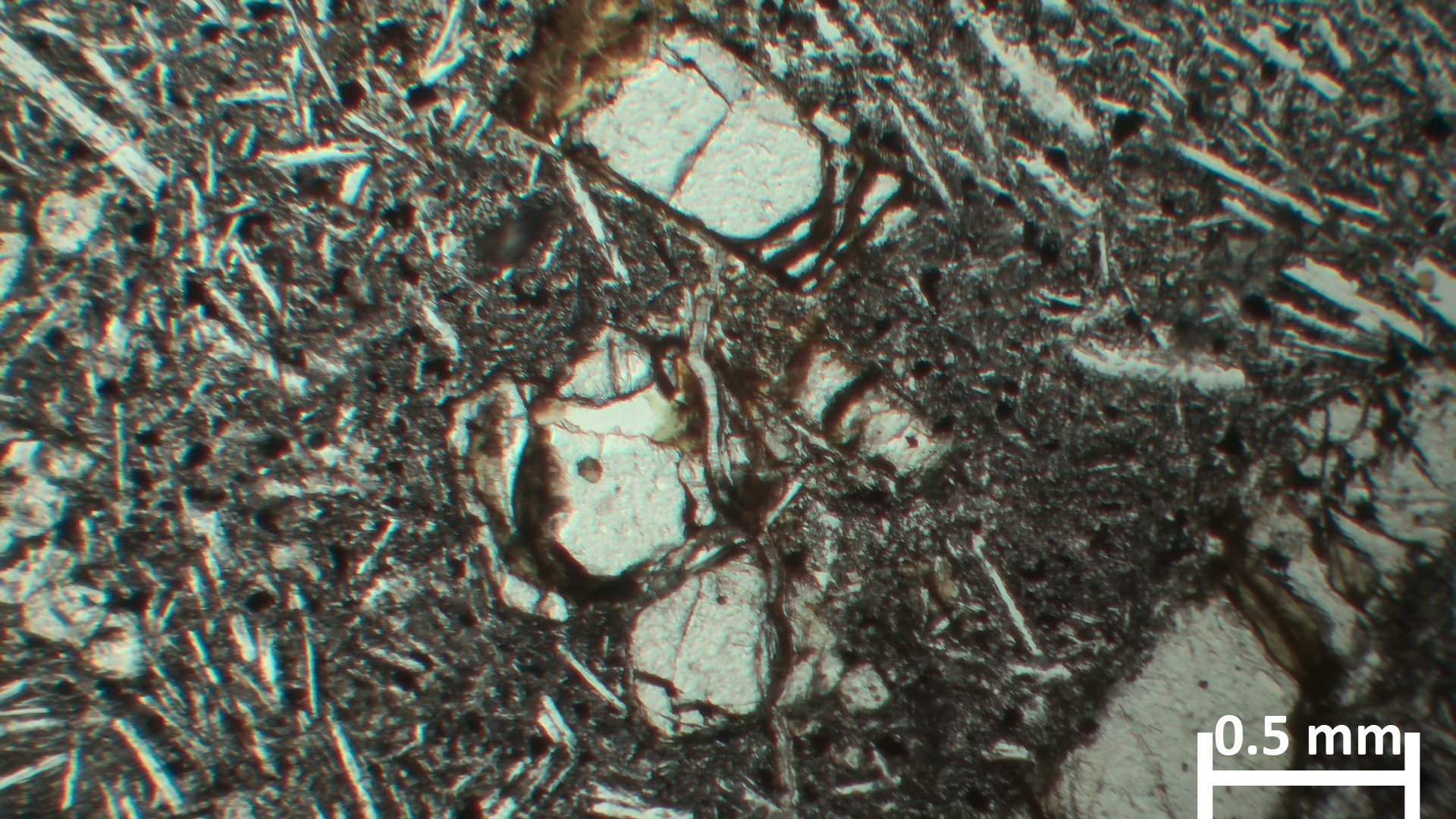


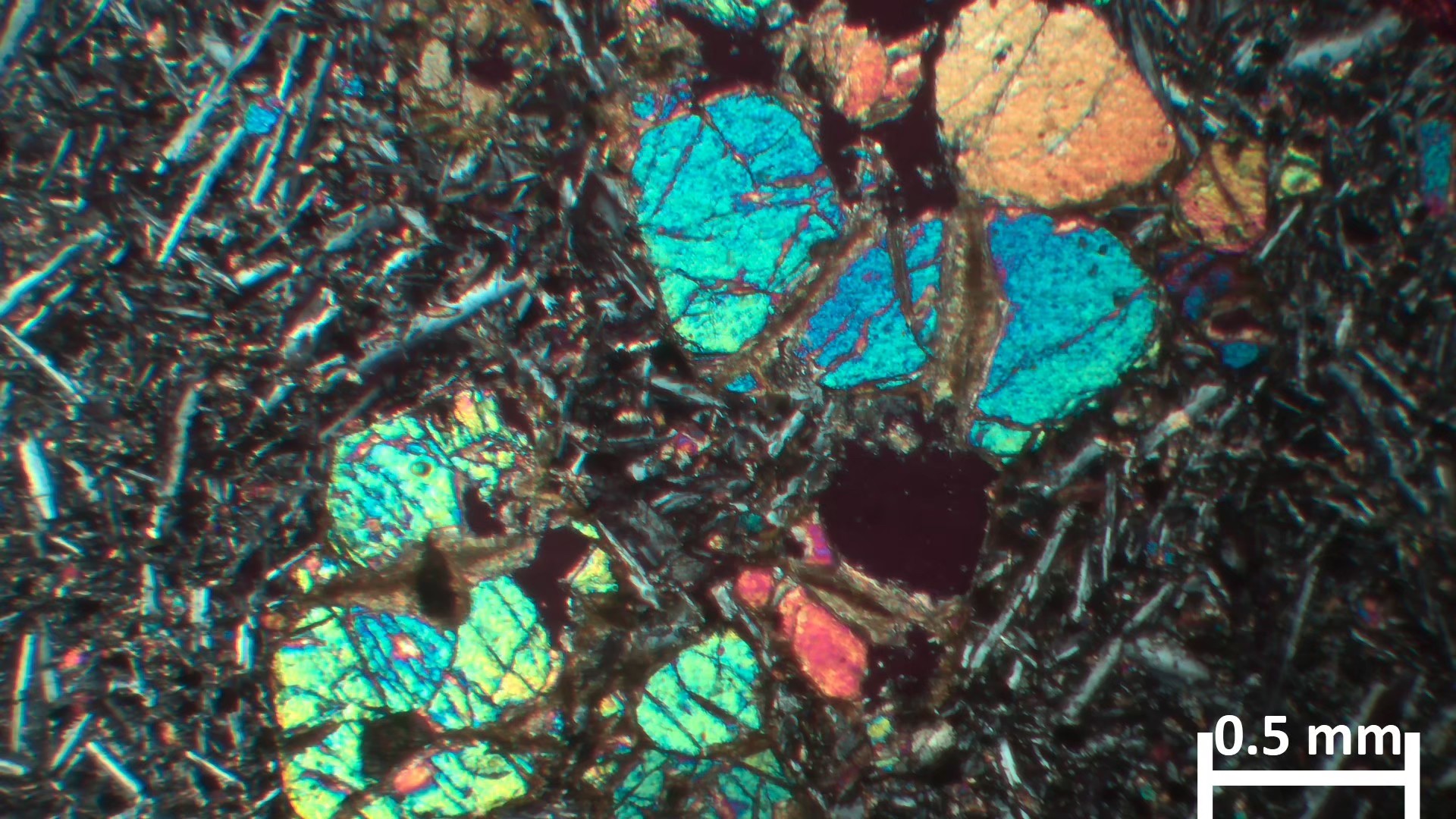


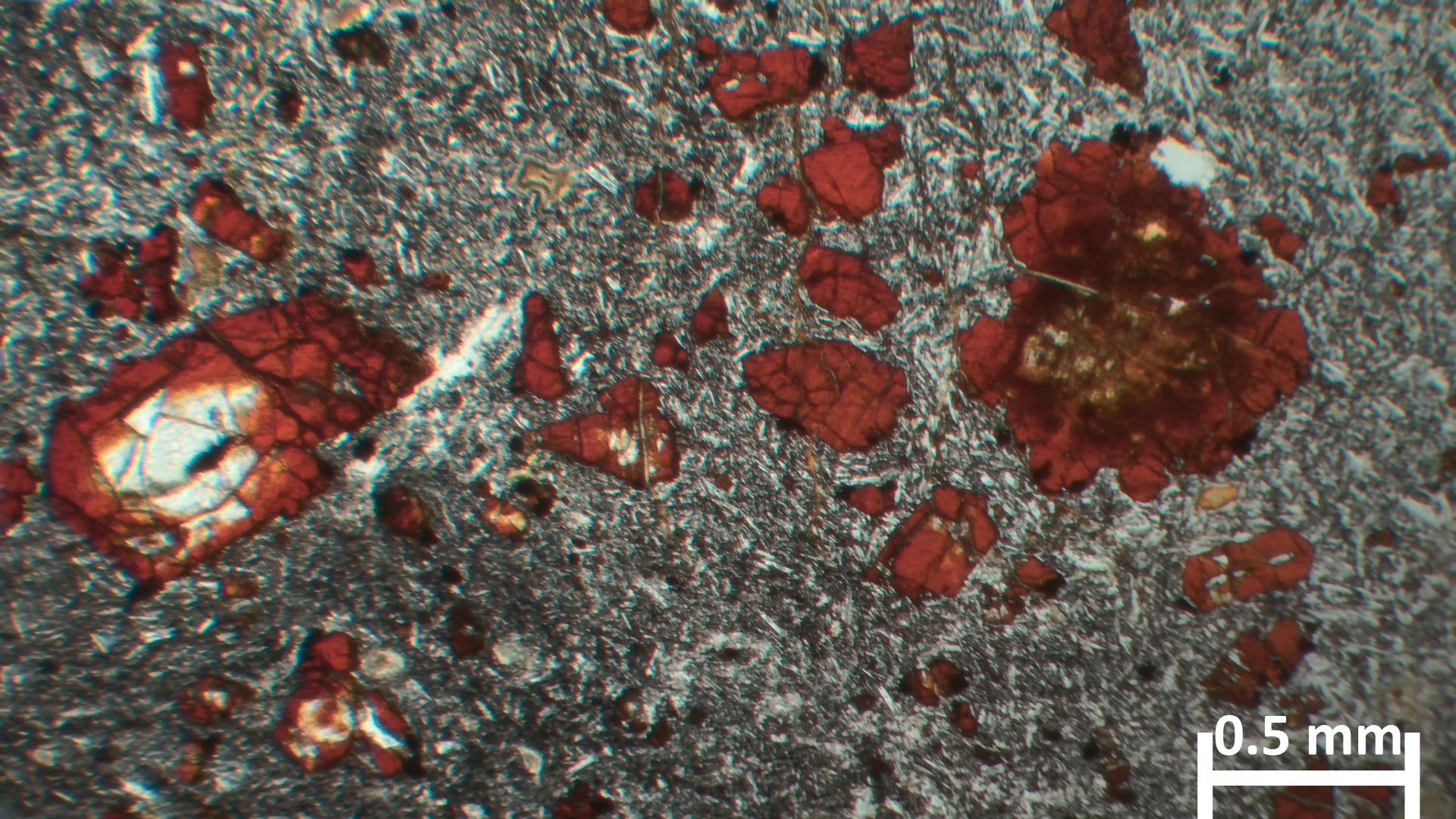


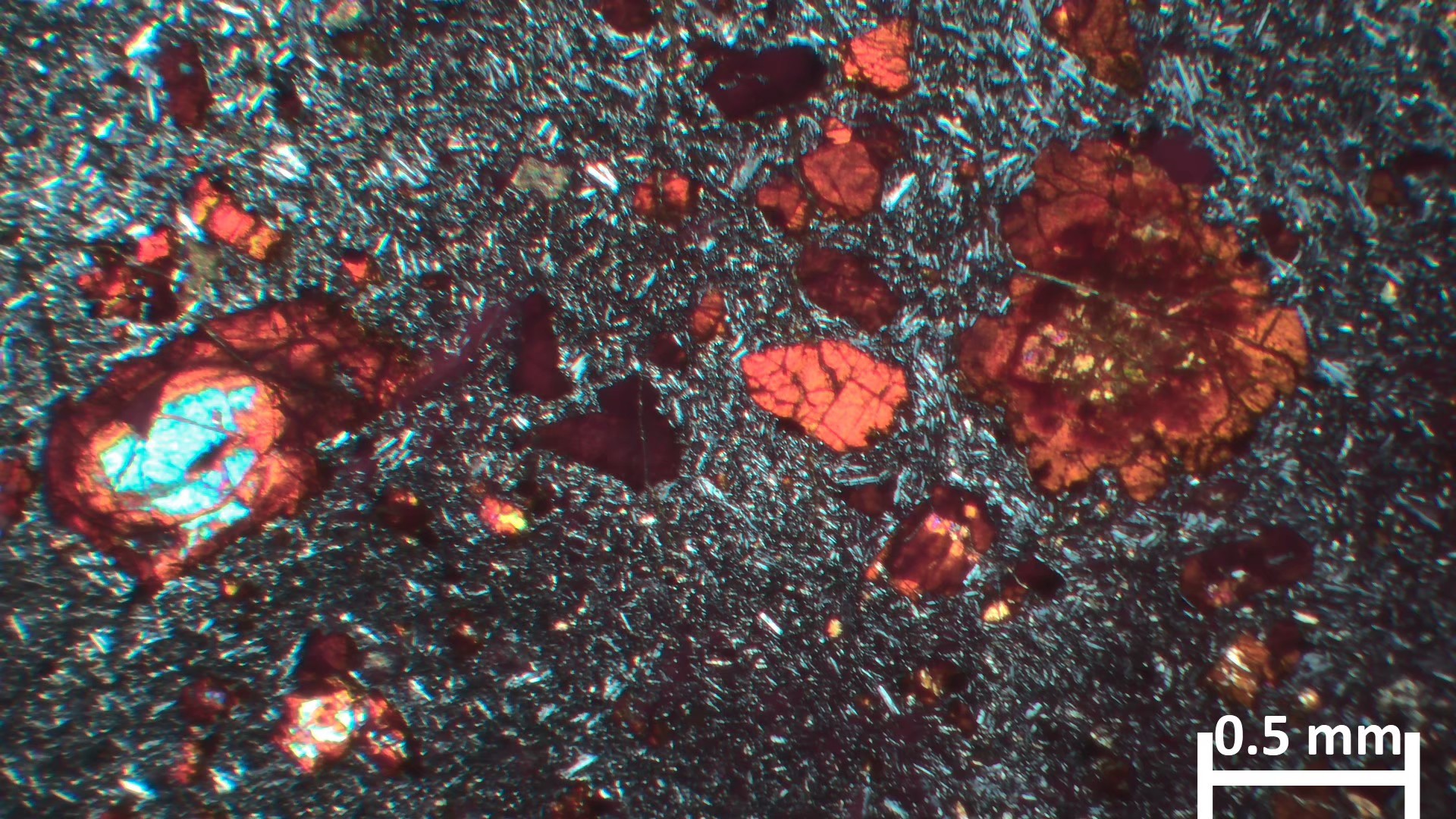


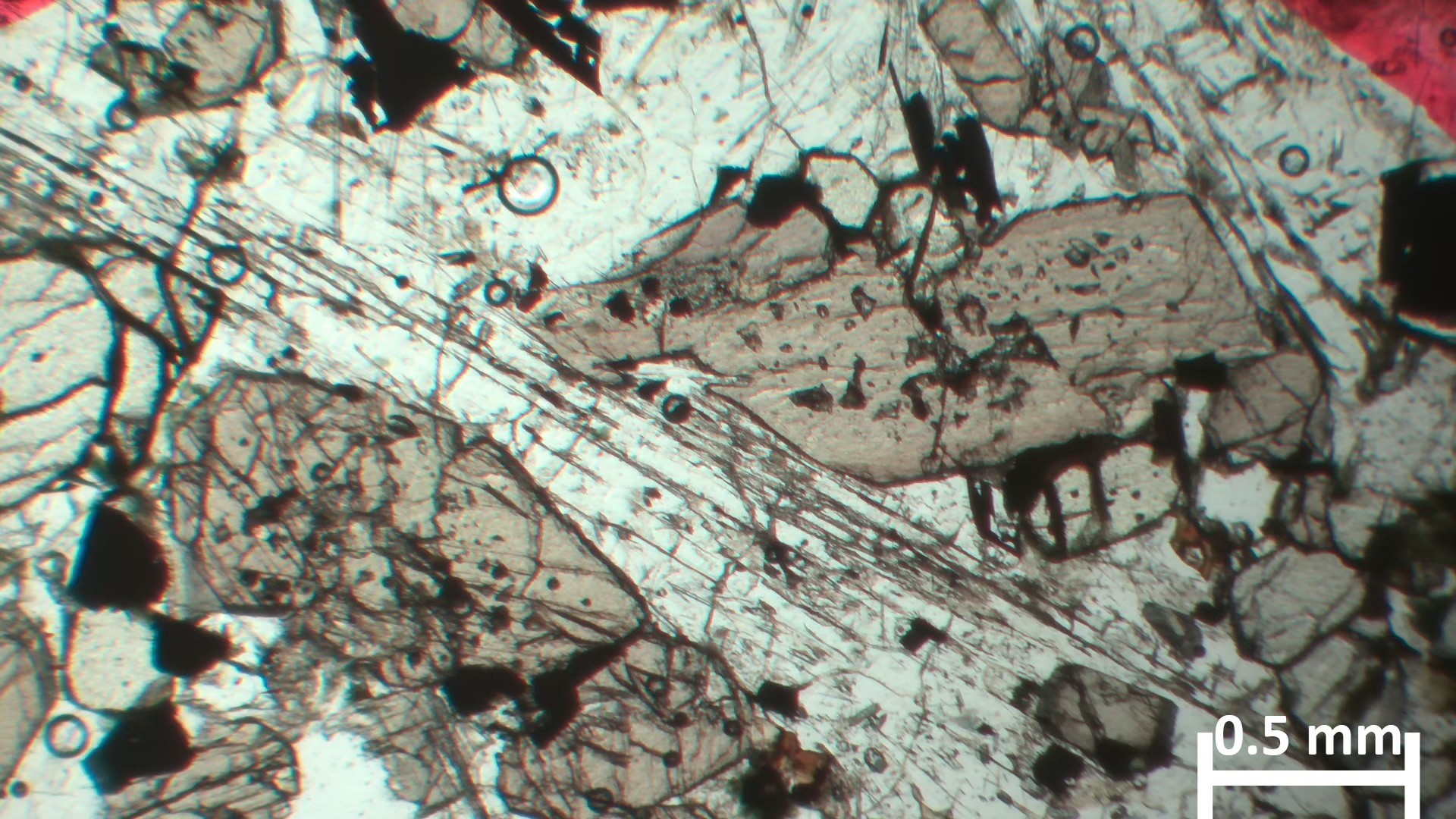


‘
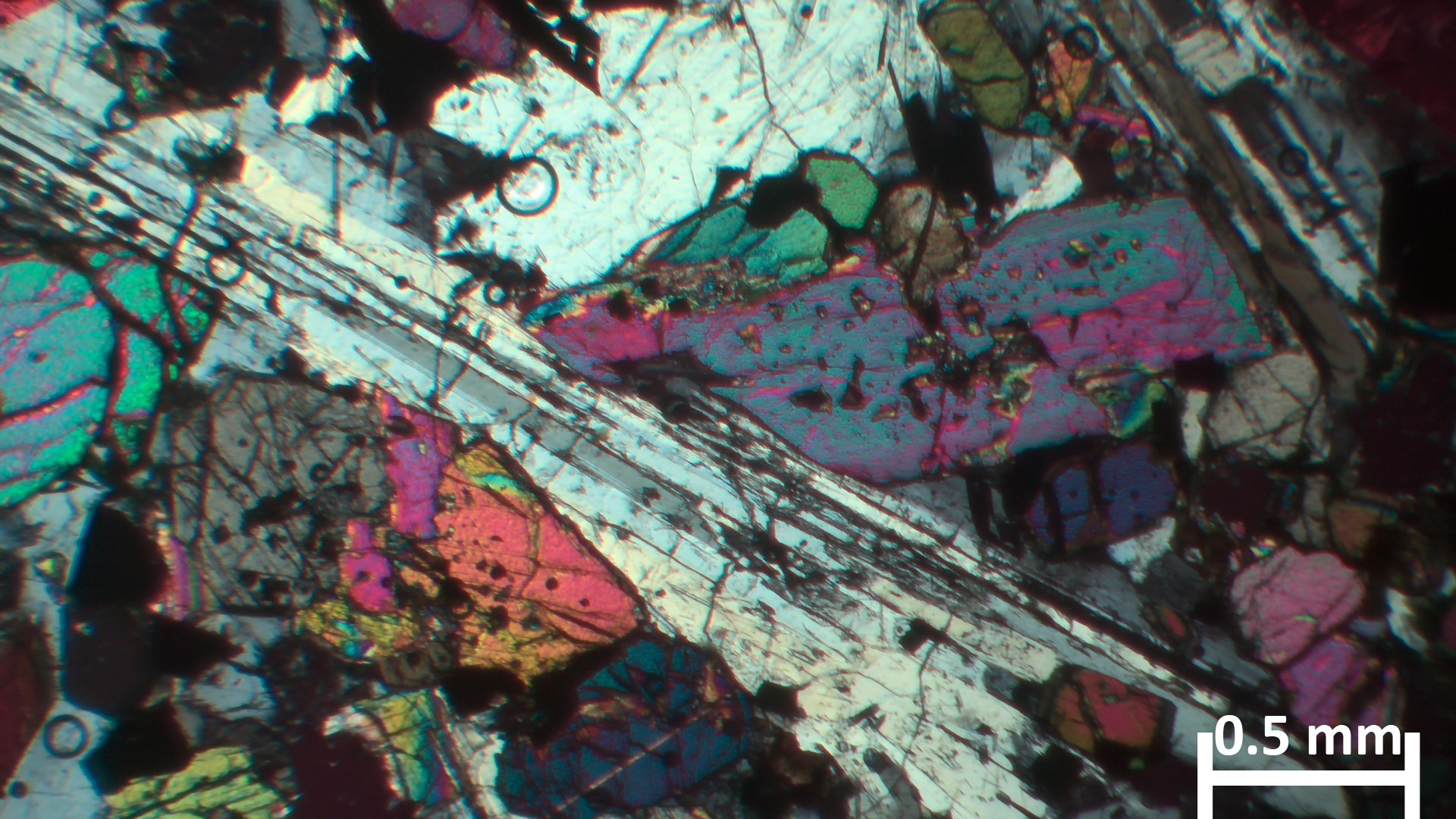


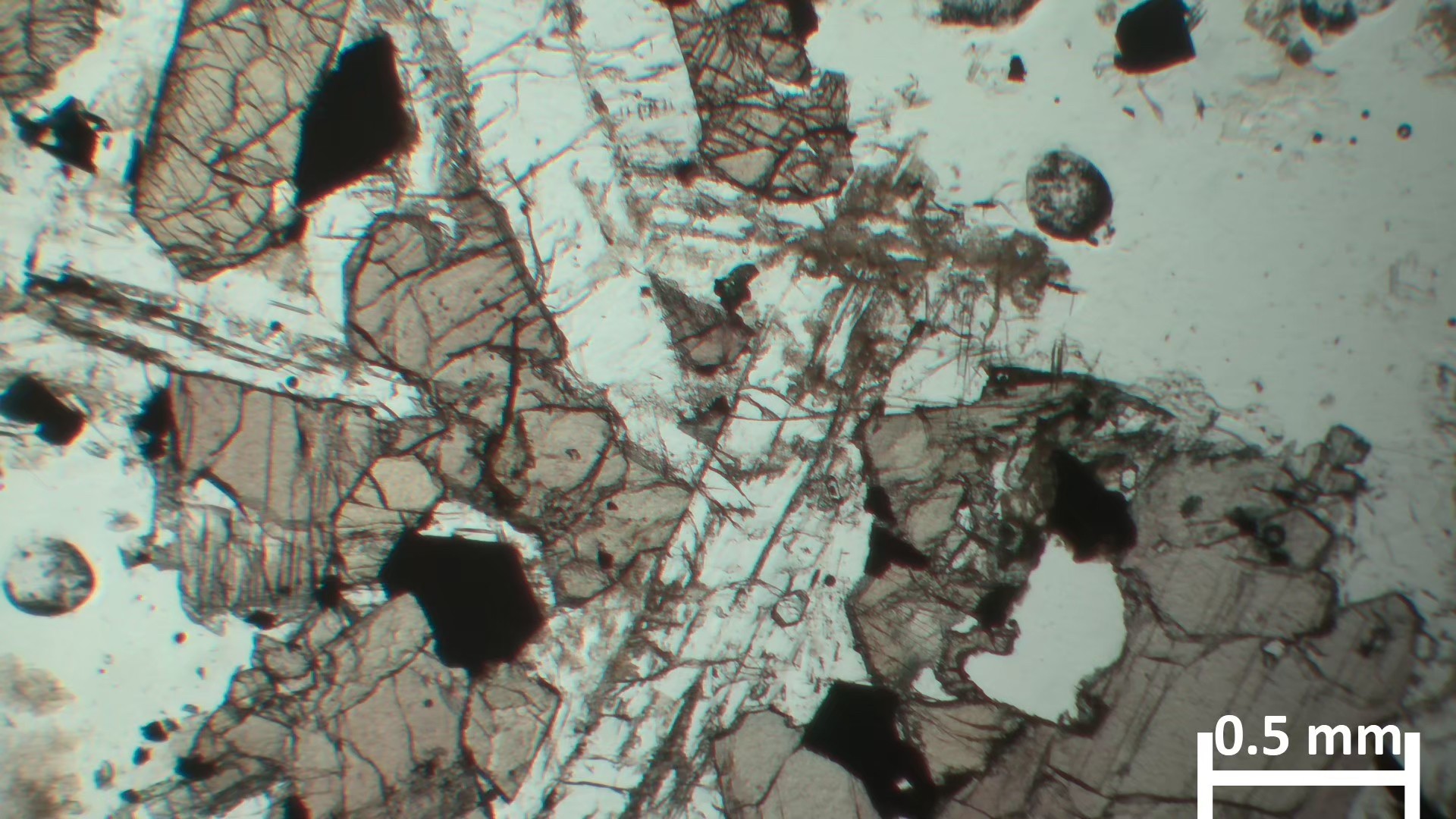


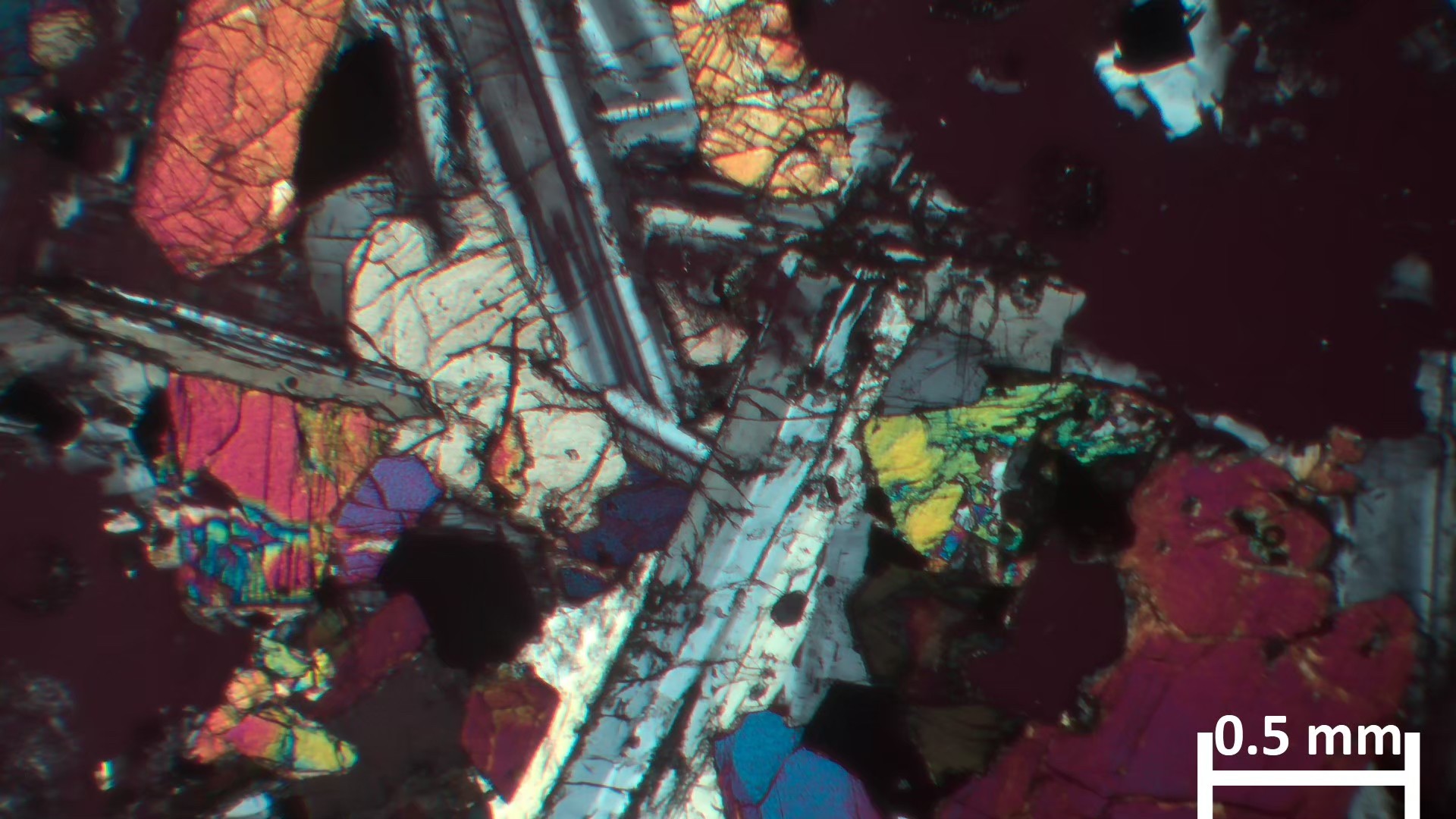


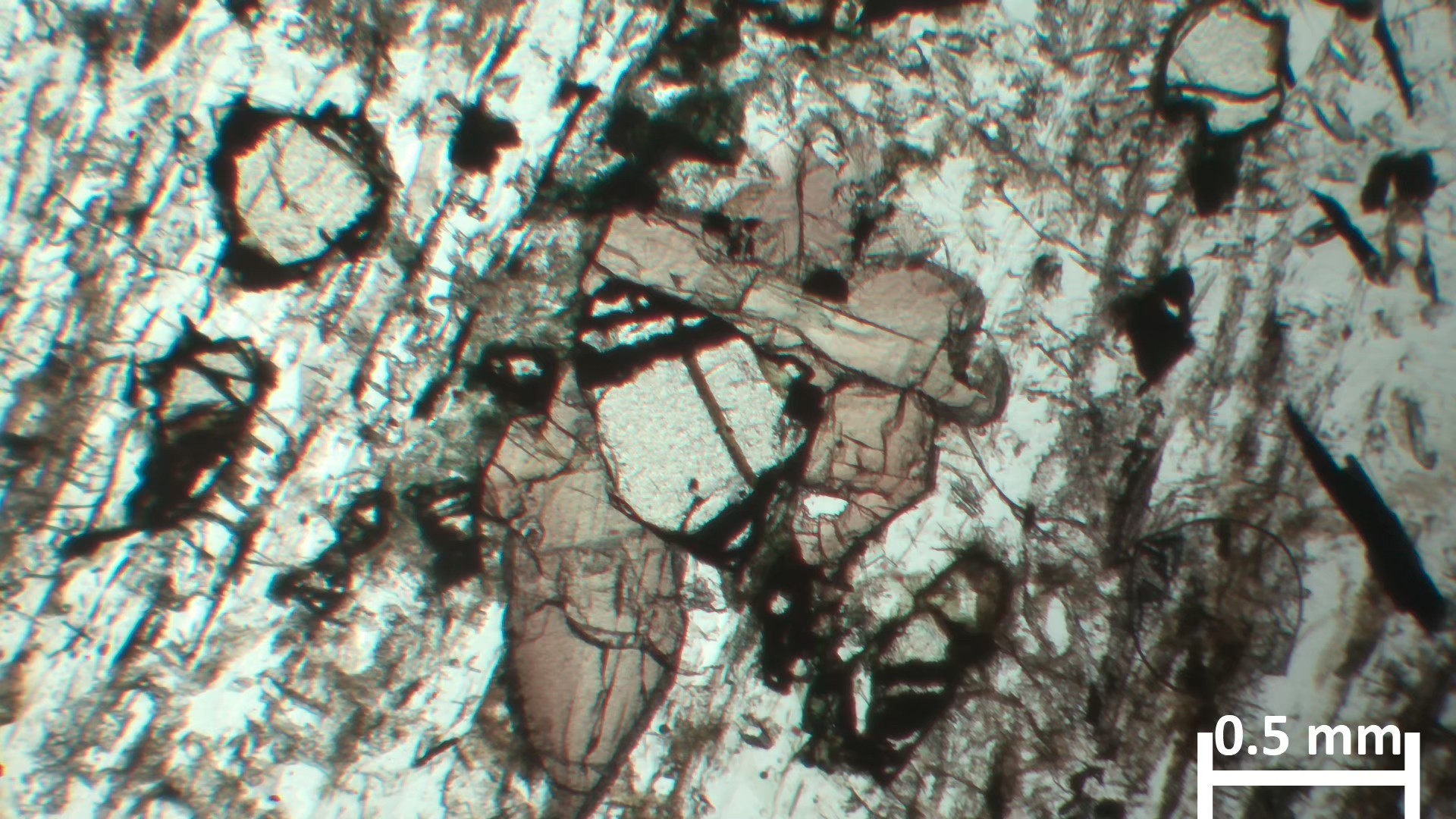


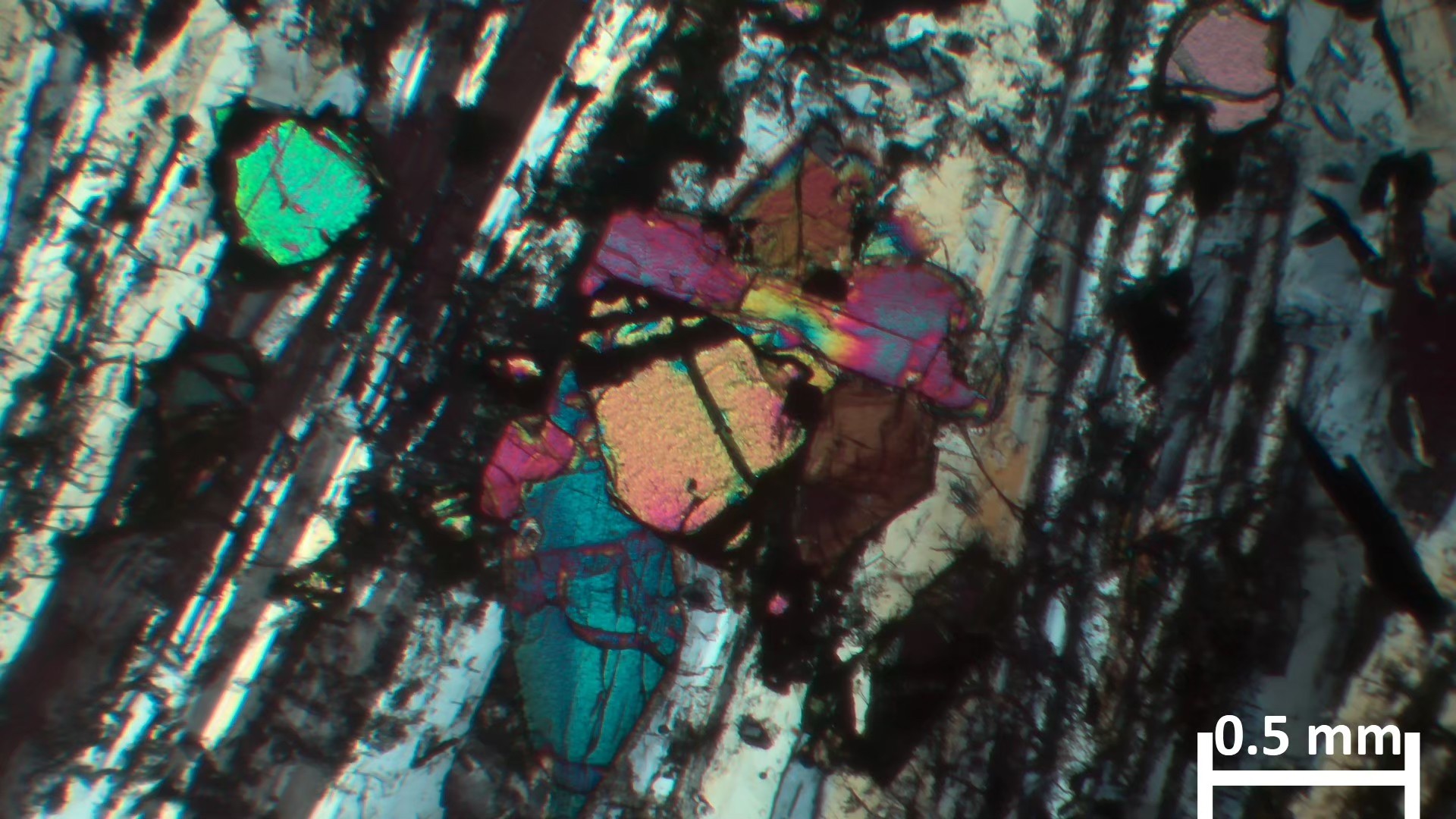


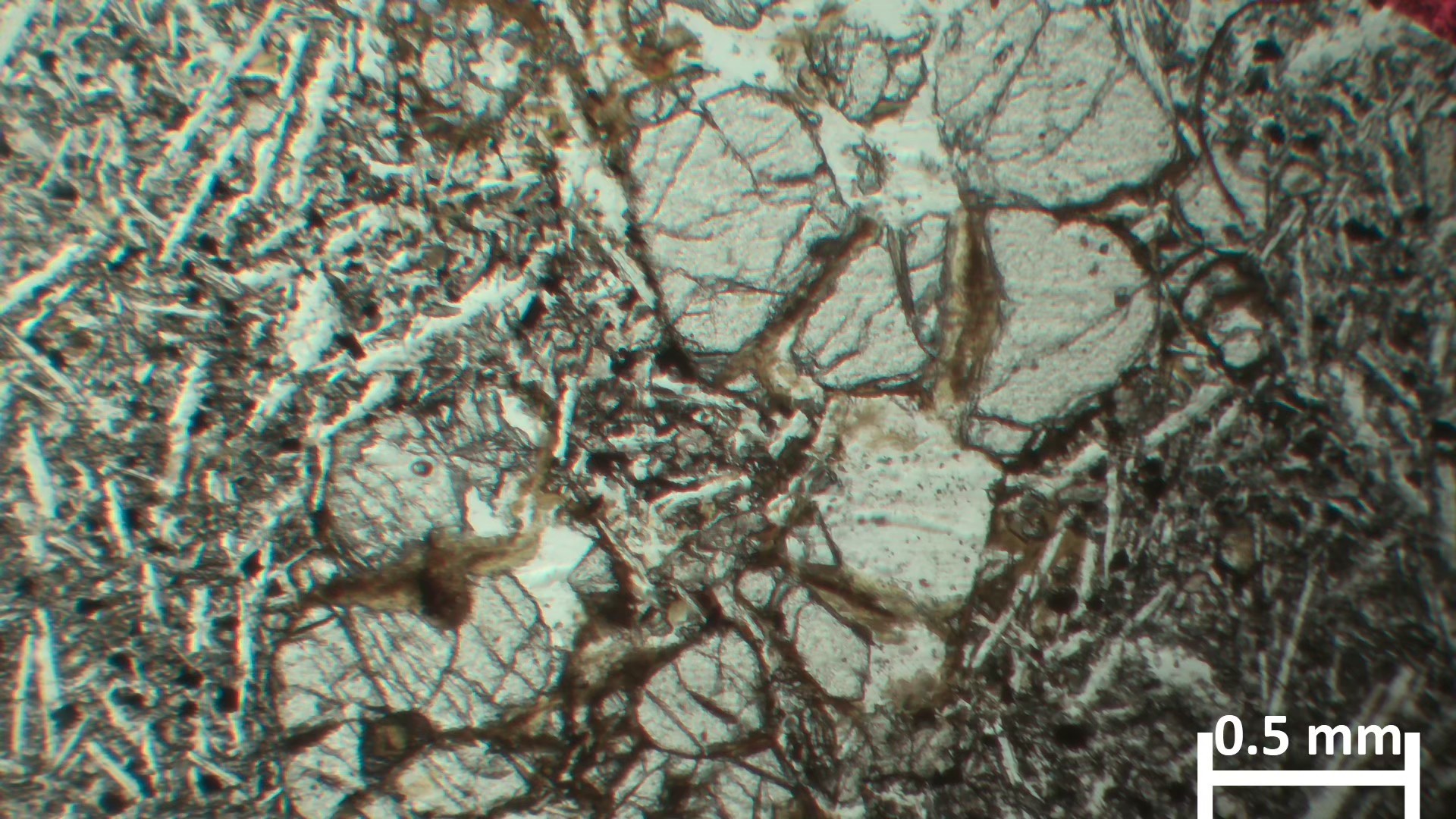


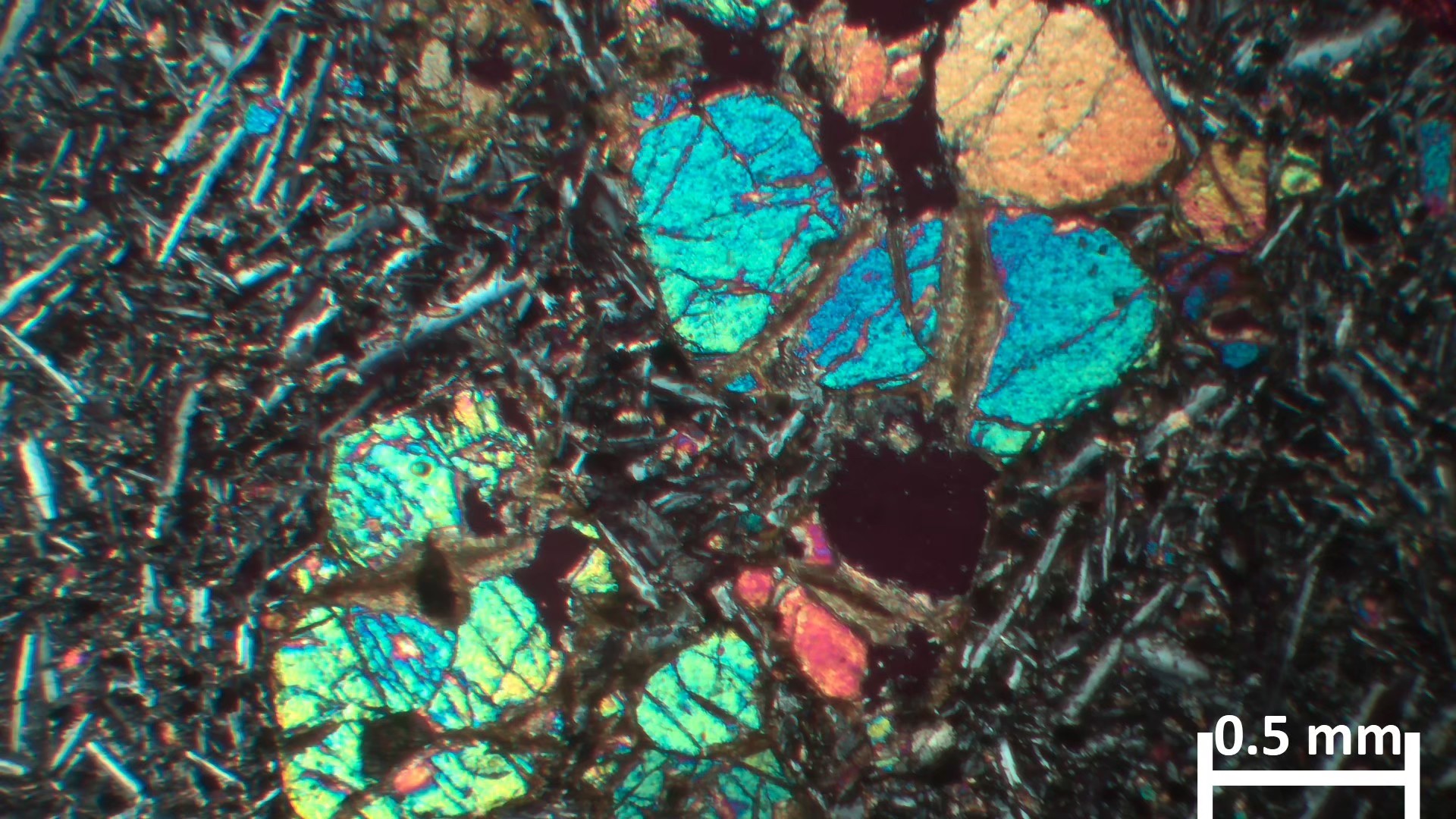


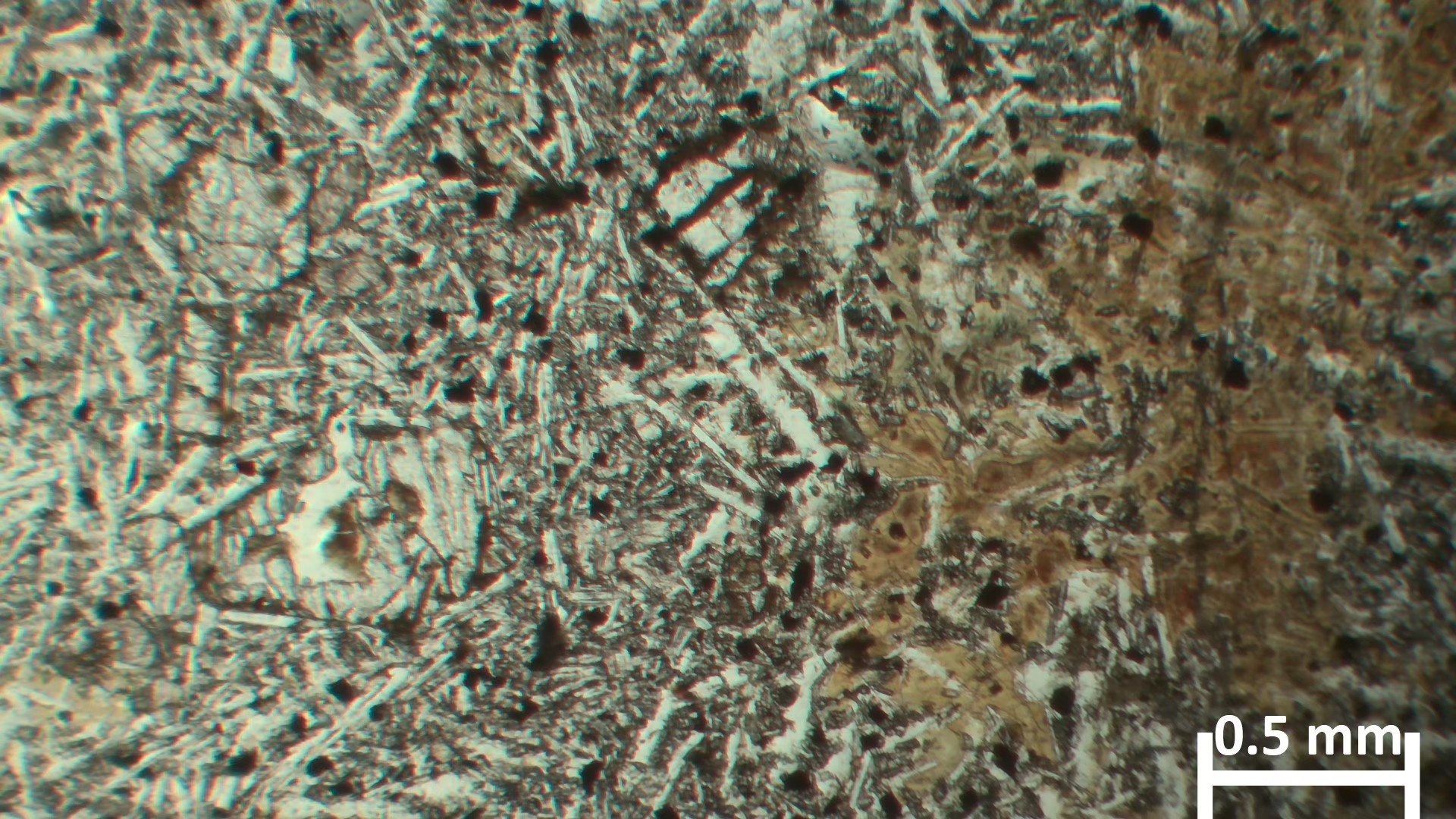


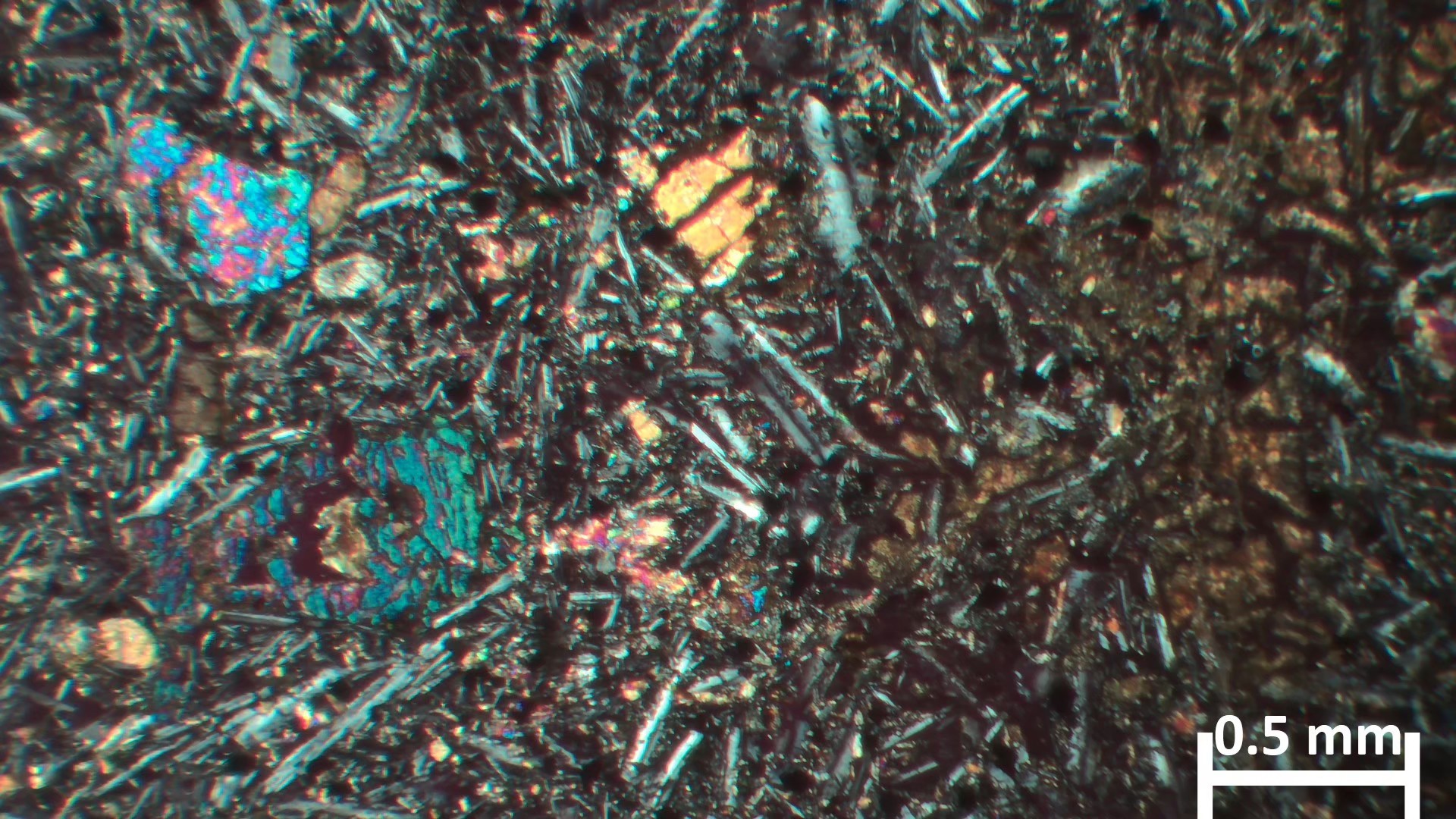


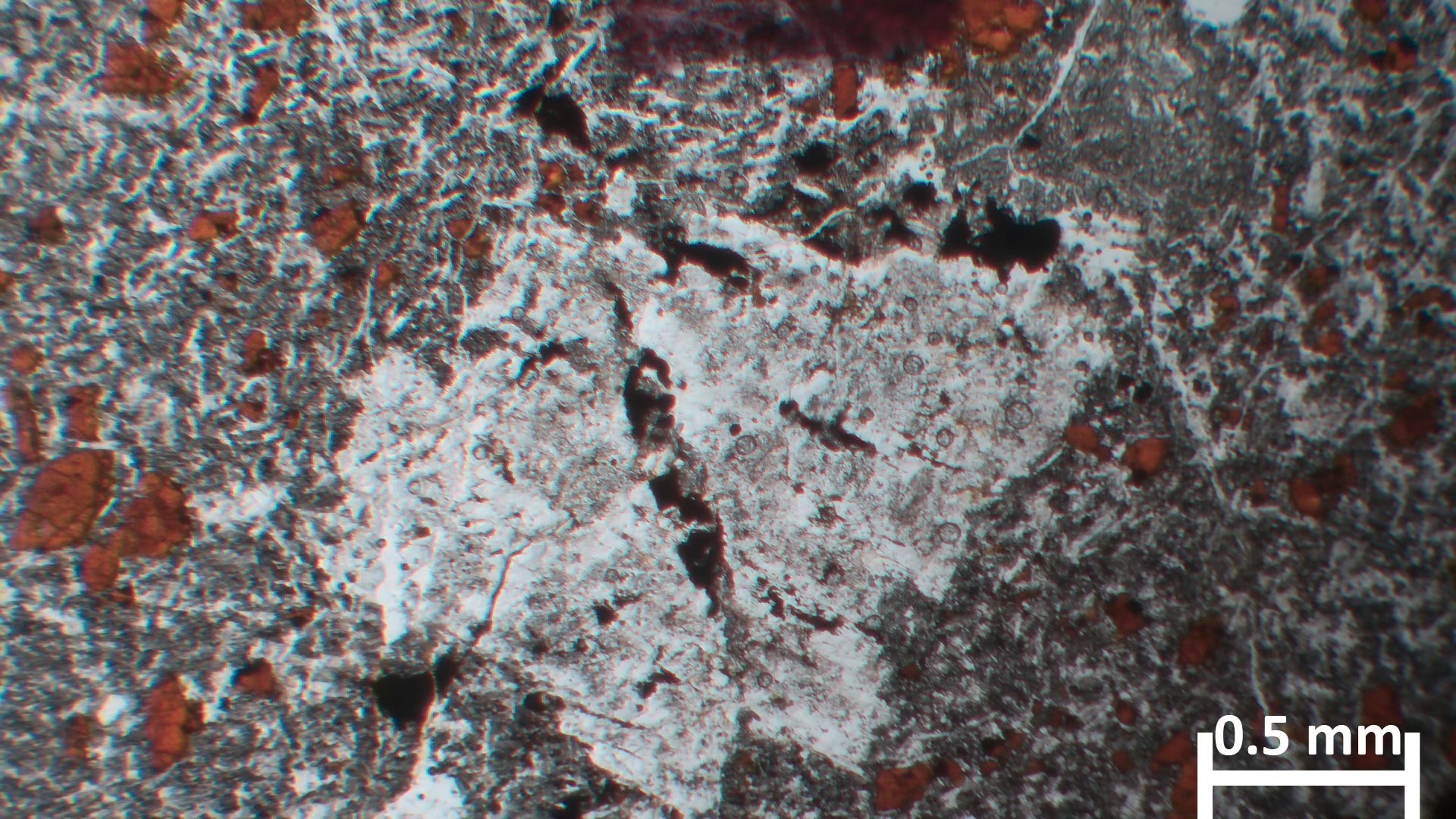


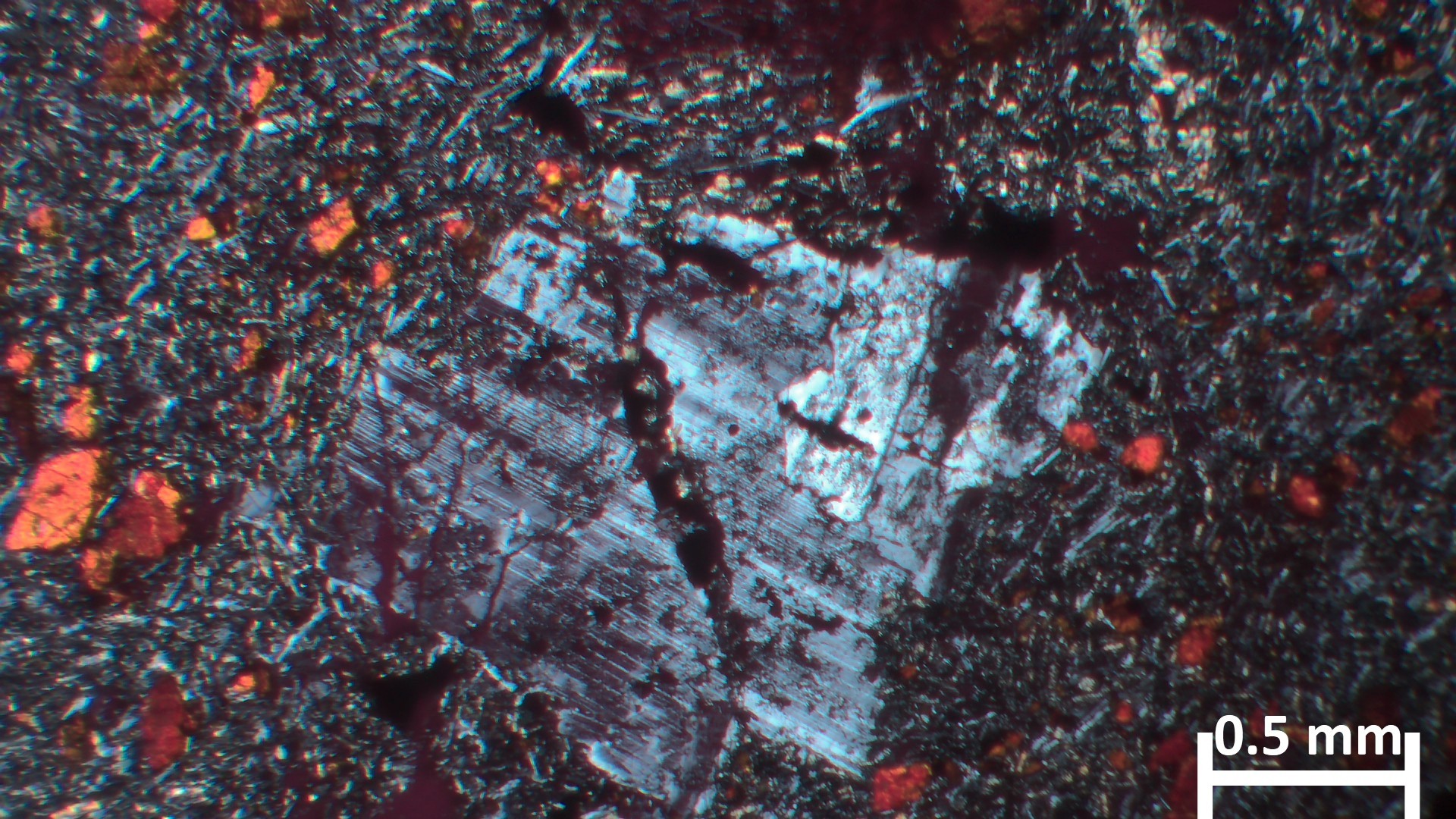


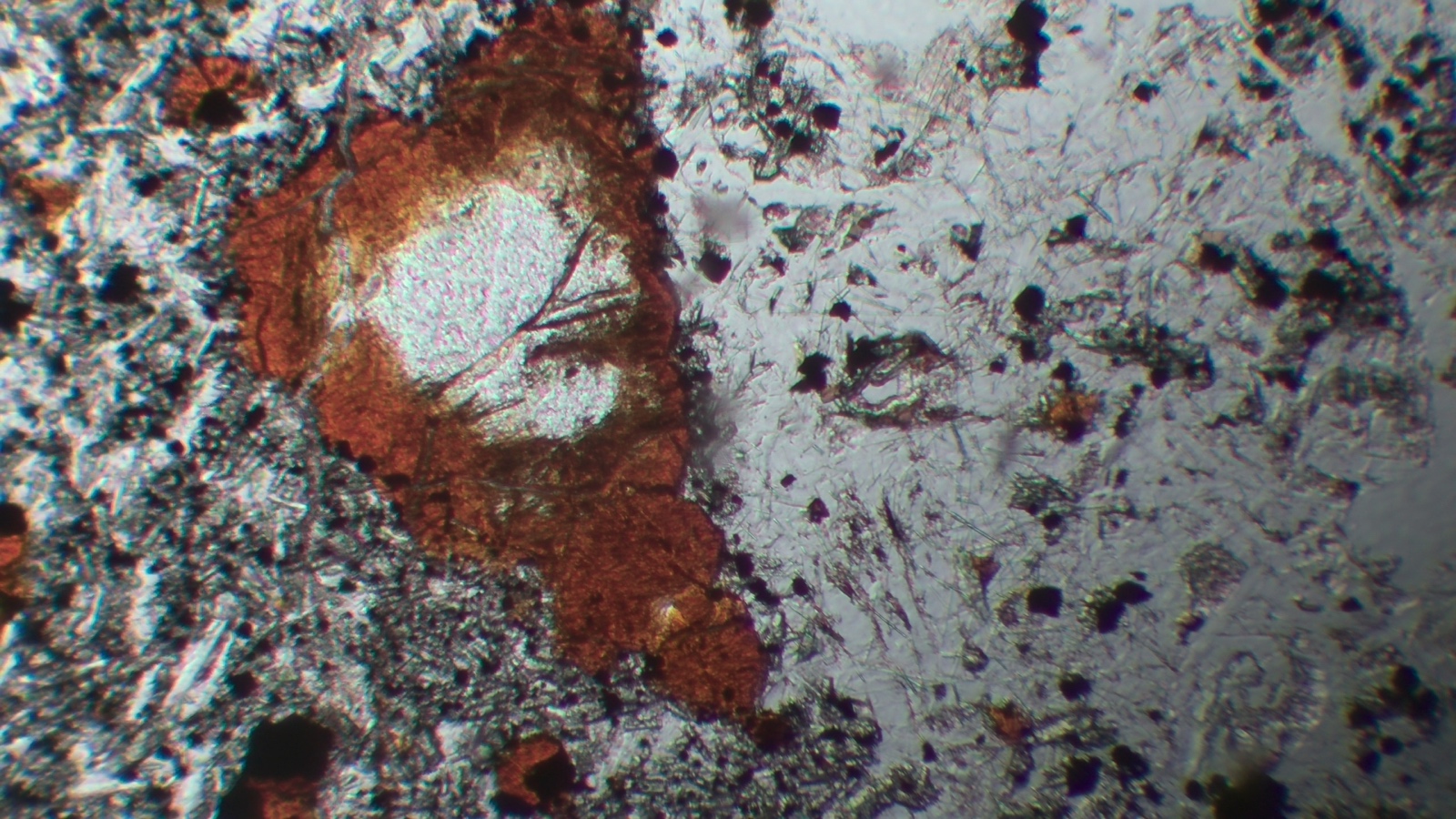


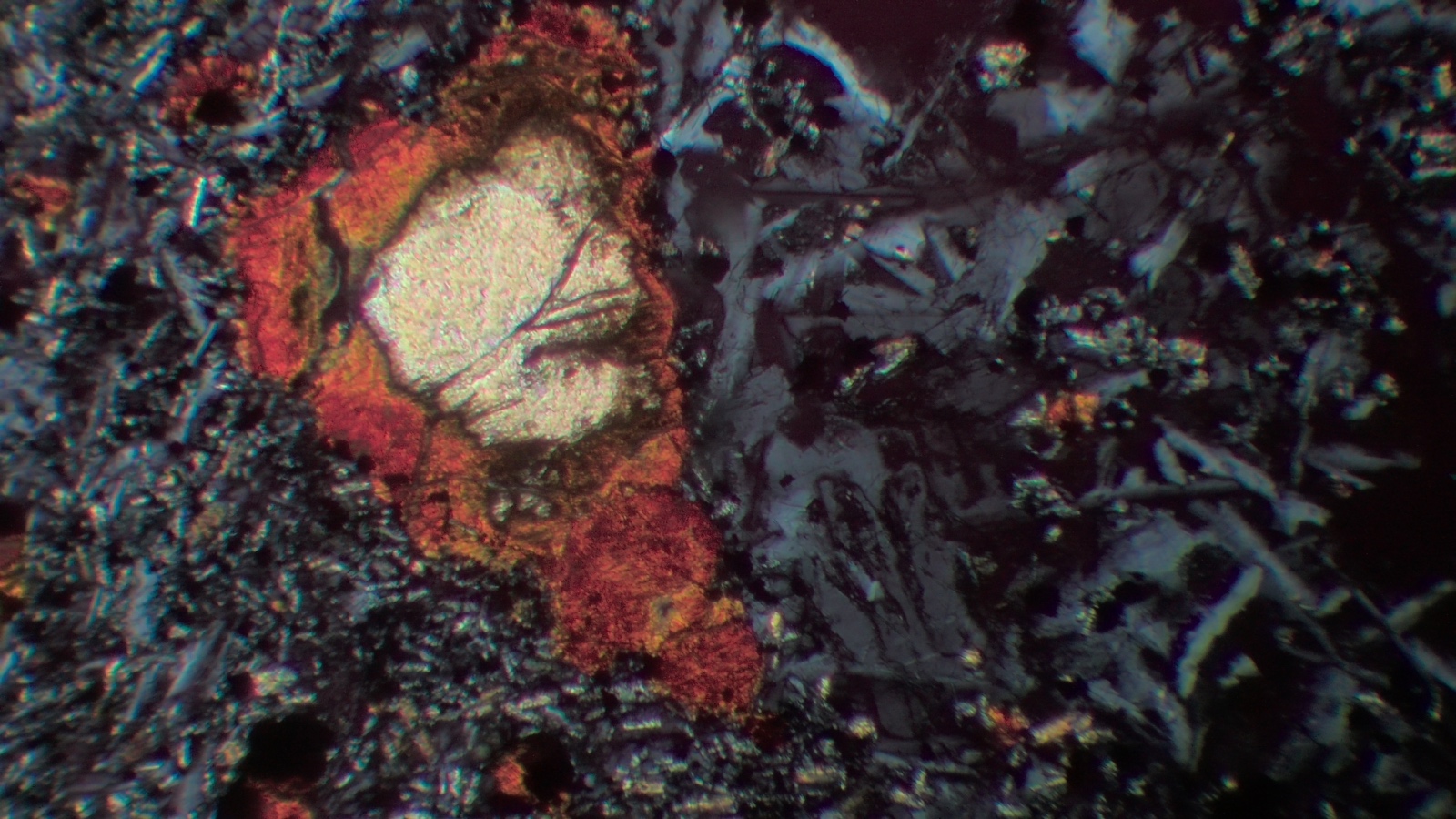


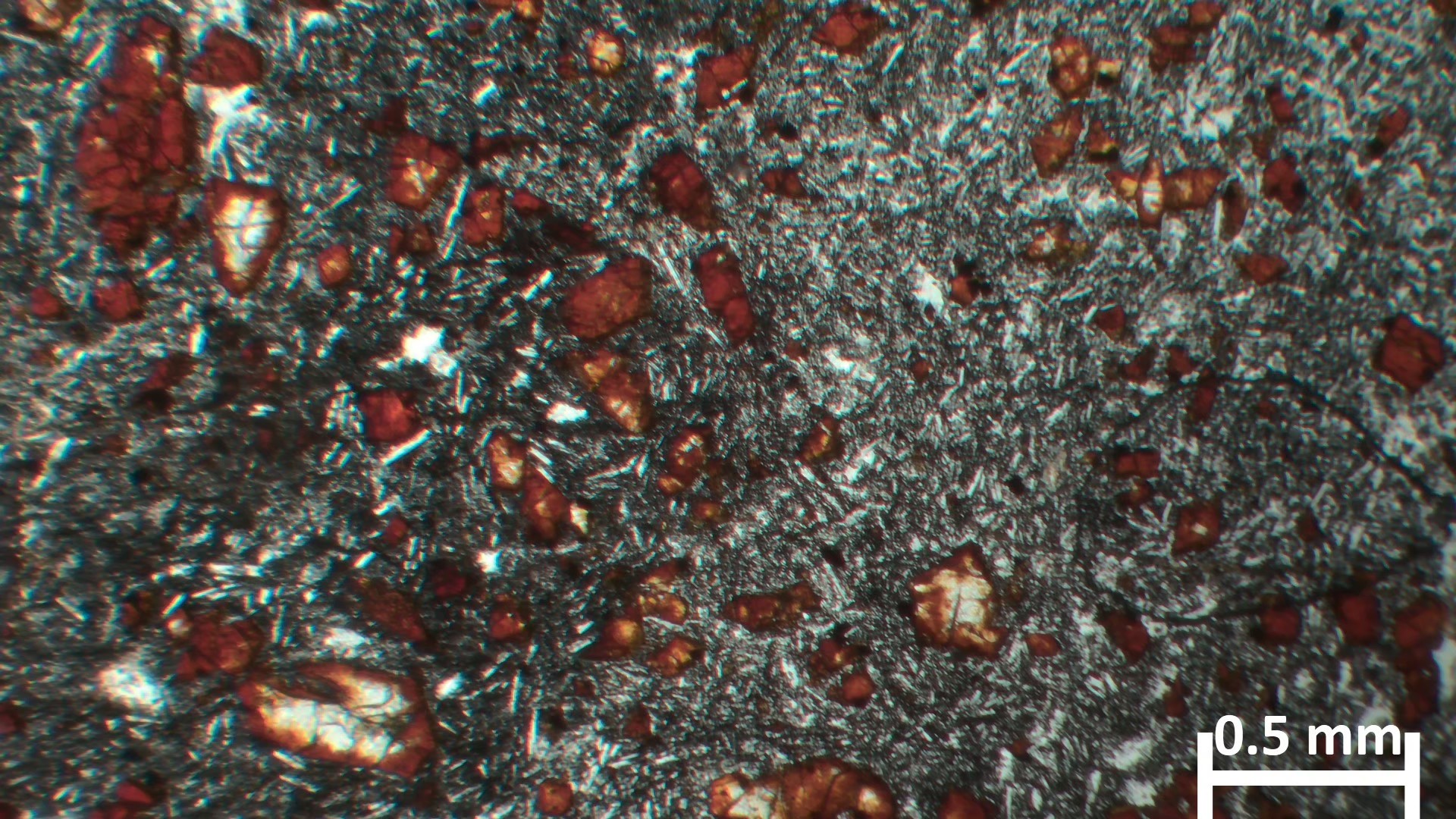


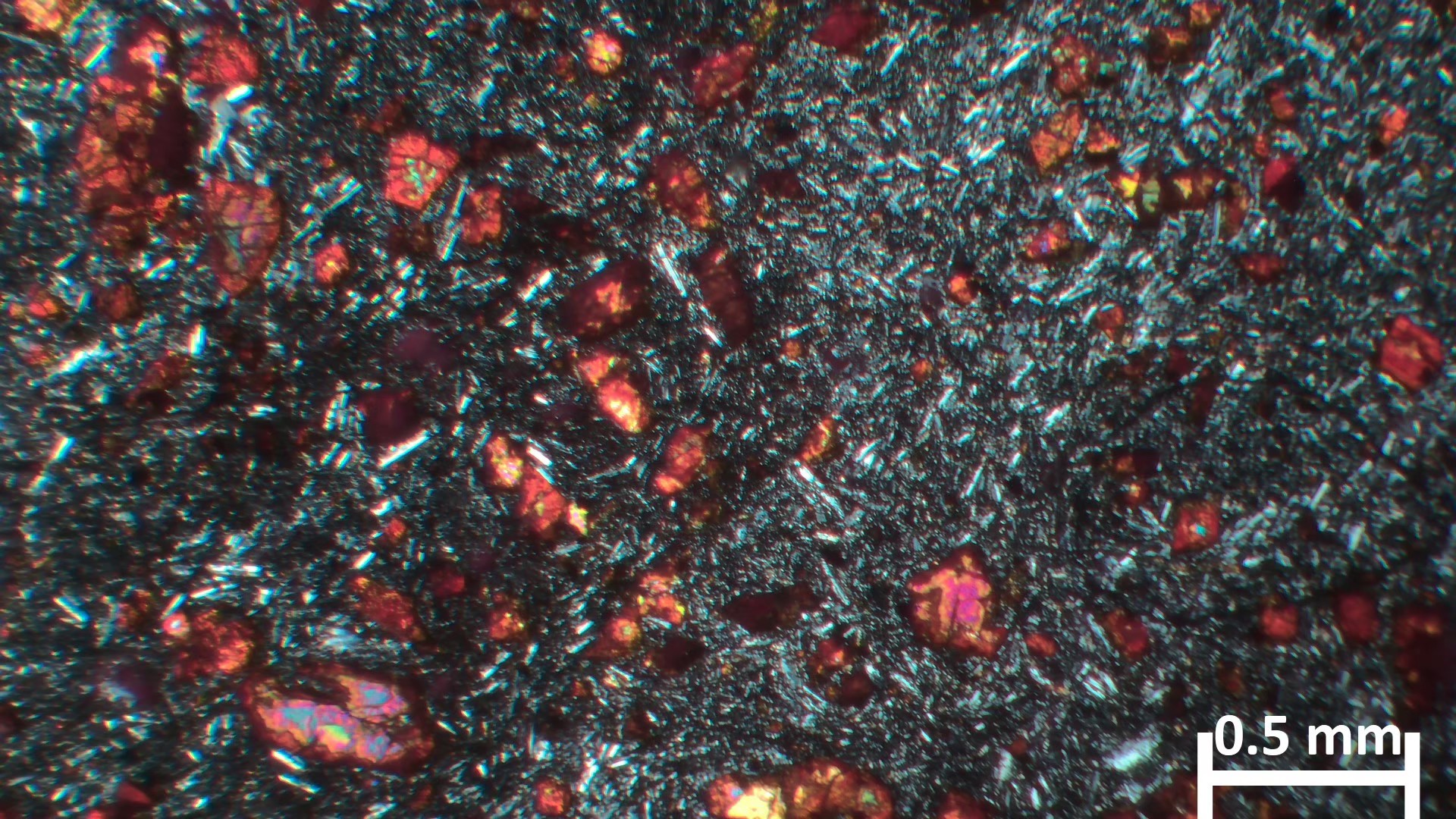

Supplement: Supplementary file 2 [file mmc2.docx]
